# Structural basis for overhang excision and terminal unwinding of DNA duplexes by TREX1

**DOI:** 10.1371/journal.pbio.2005653

**Published:** 2018-05-07

**Authors:** Kuan-Wei Huang, Tung-Chang Liu, Ruei-Yue Liang, Lee-Ya Chu, Hiu-Lo Cheng, Jhih-Wei Chu, Yu-Yuan Hsiao

**Affiliations:** 1 Department of Biological Science and Technology, National Chiao Tung University, Hsinchu, Taiwan, ROC; 2 Institute of Molecular Medicine and Bioengineering, National Chiao Tung University, Hsinchu, Taiwan, ROC; 3 Institute of Bioinformatics and Systems Biology, National Chiao Tung University, Hsinchu, Taiwan, ROC; 4 Institute of Molecular Biology, Academia Sinica, Taipei, Taiwan, ROC; 5 Chemical Biology and Molecular Biophysics Program, Taiwan International Graduate Program, Academia Sinica, Nankang, Taipei, Taiwan, ROC; 6 Institute of Bioinformatics and Structural Biology, National Tsing Hua University, Hsinchu, Taiwan, ROC; Georgia Institute of Technology, United States of America

## Abstract

Three prime repair exonuclease 1 (TREX1) is an essential exonuclease in mammalian cells, and numerous in vivo and in vitro data evidenced its participation in immunity regulation and in genotoxicity remediation. In these very complicated cellular functions, the molecular mechanisms by which duplex DNA substrates are processed are mostly elusive because of the lack of structure information. Here, we report multiple crystal structures of TREX1 complexed with various substrates to provide the structure basis for overhang excision and terminal unwinding of DNA duplexes. The substrates were designed to mimic the intermediate structural DNAs involved in various repair pathways. The results showed that the Leu24-Pro25-Ser26 cluster of TREX1 served to cap the nonscissile 5′-end of the DNA for precise removal of the short 3′-overhang in L- and Y-structural DNA or to wedge into the double-stranded region for further digestion along the duplex. Biochemical assays were also conducted to demonstrate that TREX1 can indeed degrade double-stranded DNA (dsDNA) to a full extent. Overall, this study provided unprecedented knowledge at the molecular level on the enzymatic substrate processing involved in prevention of immune activation and in responses to genotoxic stresses. For example, Arg128, whose mutation in TREX1 was linked to a disease state, were shown to exhibit consistent interaction patterns with the nonscissile strand in all of the structures we solved. Such structure basis is expected to play an indispensable role in elucidating the functional activities of TREX1 at the cellular level and in vivo.

## Author summary

Three prime repair exonuclease 1 (TREX1) was shown to participate in various cellular events such as DNA repair, immunity regulation, and viral infection. In addition to relating to autoimmune diseases, this exonuclease also acts as a potential protein target for anticancer or antiviral therapies. A key for such broad attendance of TREX1 is the activities of precise trimming of the 3′-overhang in a double-stranded (dsDNA) and breaking of the terminal base pairing of the duplex. Here, we designed a series of structural DNA substrates and activity assays to delineate the underlying mechanisms. The structures newly resolved in this work indicated that the Leu24-Pro25-Ser26 cluster of TREX1 is essential for the enzyme to carry out the aforementioned activities. Together, our results established an integrated structure view into the versatile exonuclease functions of TREX1 and illuminated the molecular origin for the unique catalytic properties of TREX1 in processing various DNA intermediates in DNA repair and in cytosolic immunity regulation.

## Introduction

Three prime repair exonuclease 1 (TREX1) is a member of the DEDDh family of exonucleases and accounts for most of the 3′–5′ exonuclease activity in mammalian cells [1,2]. Anchored in the plasma membrane of the endoplasmic reticulum (ER) through the C-terminal domain, TREX1 degrades a variety of substrates to prevent initiation of autoimmunity [3–8]. The targeted nucleic acids in this activity include single-stranded DNA (ssDNA) [3,5] and double-stranded DNA (dsDNA) [9–11]. It was also suggested that DNA/RNA hybrids are potential targets of TREX1, since the deficiencies of TREX1 demonstrate similar features of autoimmune diseases as those of RNase H2 that was known for processing the hybrid substrates [12–15]. Such genetic materials in the cytoplasm are mostly originated from replication of aberrant DNA intermediates and possibly also due to unrestrained endogenous retroelements [8,13–17].

Malfunctioning of TREX1 has thus been shown to lead to inflammation and autoimmune diseases such as inflammatory myocarditis in *Trex1*^*-/-*^ mice [18] and systemic lupus erythematosus (SLE), Aicardi-Goutières syndrome (AGS), retinal vasculopathy, cerebral leukodystrophy, and familial chilblain lupus (FCL) in TREX1-deficient humans [14,19,20]. The ability of TREX1 in modulating immune responses is also exploited by type 1 human immunodeficiency retrovirus (HIV-1) that enslaves cytosolic TREX1 to degrade its cDNA, thereby escaping detection by the nucleic acid sensors of the infected host and hence the concomitant antiviral responses [21]. Recent studies indeed showed that TREX1 knockdown in human tissues and humanized mice delayed HIV infection and suppressed local viral replication with increased production of type 1 interferons [22].

Moreover, TREX1 was shown to respond to the events of DNA damage or Granzyme A (GzmA)-mediated apoptosis with concomitant translocation to the nucleus [3,23,24]. TREX1 was indeed characterized as an exonuclease that involves in DNA repair and in DNA proofreading via working with other nuclear enzymes such as Poly [ADP-ribose] polymerase 1 (PARP-1) and DNA polymerase β [1,24,25]. Additionally, TREX1 was established to play a role in GzmA-mediated cytotoxicity [23]. Cytotoxic T lymphocytes and natural killer cells secrete GzmA proteases to induce cell death of the targeted cancer cells or virus-infected cells by releasing TREX1 and nonmetastatic protein 23 homolog 1 (NM23-H1) from ER-bound patient SE translocation protein (SET) complex, and their translocation to the nucleus promoted chromosomal DNA fragmentation [23]. With such diverse activities toward a variety of nucleic acid substrates—including ssDNA, dsDNA, DNA/RNA duplexes, structural DNAs, and DNA hybrids with an interject nick—the linkage of TREX1 to apoptotic DNA degradation in dying cells [26] and in chromosomal fragmentation during telomere crisis [27] was hence not unexpected. Regulation of the exonuclease activity of TREX1 was indeed recognized as an influential factor in cancer therapy [28–30].

The broad attendance of TREX1 in the activities of immune silencing and responding to genotoxic stresses highlights the elementary role of properly processing the nucleic acids that are involved in these vital yet very complicated cellular functions. Mechanistic understanding of substrate handling is hence key to comprehending the connections between different enzymes in the pathways. However, such knowledge is mostly elusive as informative molecular details are lacking. For example, precise excision of short 3′-overhangs in generation of blunt-end dsDNAs and the terminal unwinding of these duplexes for further degradation are two events in urgent need of structure basis. Although X-ray structures of TREX1 with ssDNA [7,31] and Y-structural DNA are available [10], the 3′- and 5′-overhang of the substrates therein were rather long (>4 nt). Therefore, the structure information is currently insufficient to deduce the mechanism by which double-stranded substrates are processed (see Discussion).

TREX1 is, in fact, unique in the DEDDh family for the activity of dsDNA degradation, which enables its participation in preventing autoimmune responses and in restricting retrotransposons [9–11]. The DEDDh family, also named DnaQ-like exonuclease family, or RNase T superfamily in the protein families (Pfam) database (https://pfam.xfam.org/), contains over 17,000 members across more than 3,000 species [32,33]. Toward ssDNA substrates, the catalytic properties of TREX1 are similar to those of the *Escherichia coli* homolog, RNase T. When first reported, both enzymes were shown to exhibit activities involved in DNA repair, namely trimming the single-stranded segments of the mispaired ends in a DNA duplex, such as the single-stranded 3′-overhangs in duplex, Y-, or flap structural DNAs [1,34,35]. The analogy in this activity is in line with the in vivo and in vitro experiments that concluded that the two nucleases likely play similar roles in various pathways of DNA repair [24,33]. Furthermore, TREX1 can digest single-stranded RNA (ssRNA) as RNase T does, and this RNase activity of TREX1 is potentially responsible for degrading DNA/RNA hybrids in the cytosol [13,14] and/or during tRNA maturation [36]. The main difference between TREX1 and RNase T, though, is the catalytic ability of TREX1 in degrading dsDNA, for which RNase T lacks [35,37].

In this work, we solved 4 X-ray structures of TREX1 with representative DNA substrates to delineate the molecular details of the multifaceted catalytic properties of TREX1. On one hand, TREX1 allows precise trimming of structural DNAs to generate blunt-end products in preparation for specific pathways of DNA repair. On the other hand, the enzyme enables digestion of dsDNA for immunity regulation. The 4 new structures we presented here reveal how TREX1 removes the single-stranded 3′-overhang in producing DNA duplexes with blunt ends and, for the first time, illustrate how the enzyme overcomes the resistance of the double-strand structure for degradation of dsDNA. The structural DNA substrates in our analysis were also specifically designed to mimic the DNA intermediates in various DNA repair pathways. Therefore, the new structures presented in this work provide unprecedented insights into the functional roles of TREX1 at the molecular level. Together with measurements of nuclease activities, the structure-based mechanism was also employed to discuss the cellular functions of TREX1 in DNA repair and in immune silencing.

## Results

**Fig 1:**
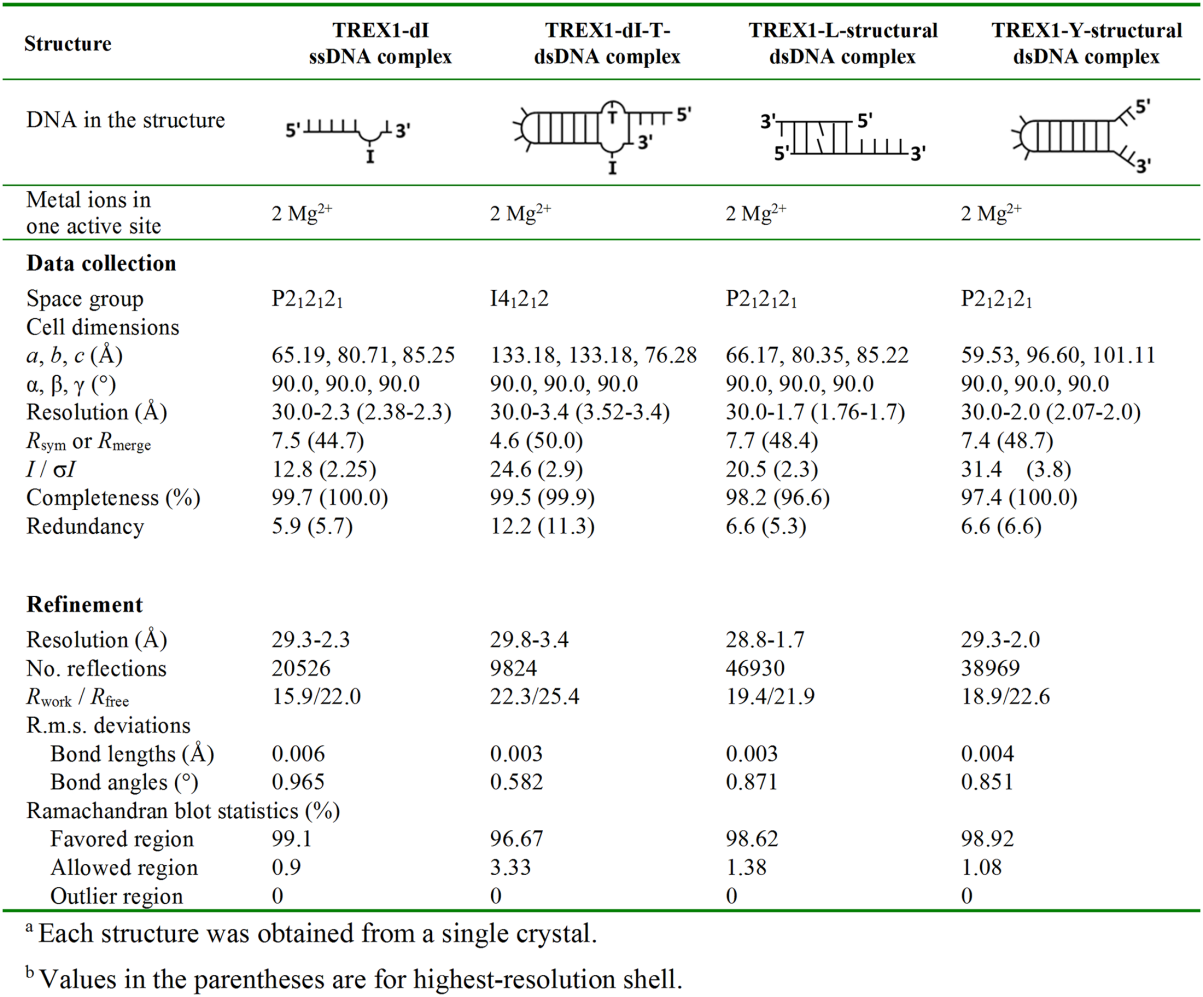
X-ray data collection and refinement statistics for TREX1-DNA complexes. dI, deoxyinosine; dsDNA, double-stranded DNA; Mg^2+^, magnesium ion; r.m.s, root-mean-square; ssDNA, single-stranded DNA; TREX1, three prime repair exonuclease 1.

We determined the structures of TREX1 complexed with 4 different substrates: (i) the TREX1-deoxyinosine (dI)-ssDNA structure at the 2.3 Å resolution is with a ssDNA containing a dI (dI-ssDNA); (ii) the TREX1-dI-T-dsDNA structure at the 3.4 Å resolution is with a dsDNA with a scissile strand containing a dI (dI-T-dsDNA); (iii) the TREX1-L-structural dsDNA structure is with a duplex DNA that the two termini of 1-nt- and 4-nt-long 3′-overhang form an S-Tetromino shape (referred to as L-structural dsDNA based on the shape of terminals); and (iv) the TREX1-Y-structural dsDNA structure is with a Y-structural dsDNA that the 3′- and 5′-ends of noncomplementary sequences form a Y-shape structure (Y-structural dsDNA). The crystallization conditions and the diffraction and refinement statistics of these structures are listed in Fig 1 and S1 Table. The crystals of these structures were all grown using mouse TREX1 with the C-terminal transmembrane domain truncated.

Nuclease activity assays showed that the exonuclease activity of TREX1 in the crystallization conditions of TREX1-dI-ssDNA, TREX1-dI-T-dsDNA, and TREX1-Y-structural dsDNA was inhibited, and the nucleic acid substrate in these structures was thus intact. The dsDNA of the TREX1-dI-T-dsDNA structure was further labeled with γ-^32^P and analyzed using 20% denaturing polyacrylamide gels to confirm that dI-T-dsDNA was indeed intact in the crystal (S1A and S1B Fig). The nuclease activity of TREX1 was enhanced in the crystallization conditions of the TREX1-L-structural dsDNA structure. In this case, the input DNA substrate was digested by TREX1 into 2 small ssDNA fragments, including 6-nt- and 9-nt-long ssDNA (6-nt-long ssDNA: 5′-GGCCCT-3′ and 9-nt-long ssDNA: 5′-GGCCCTCTT-3′). The 2 ssDNA strands then hybridized to form a duplex DNA with 1-nt- and 4-nt-long 3′-overhang in the crystal. Two magnesium ions (Mg^2+^) were observed at the TREX1 active site in all of these structures, even though that magnesium was not additionally added in preparing the crystallization conditions. These Mg^2+^ were likely from the expression hosts as similar phenomena had been observed in other structures of DEDDh exonucleases [35,37]. In the following, we first present the results of activity assays to characterize the catalytic properties of TREX1 against various substrates. Next, we discuss the 4 structures that collectively reveal the molecular principles for handling such diverse substrates.

### TREX1 can digest ssDNAs and dsDNAs containing a damaged base

Two members of DEDDh exonucleases, *Thermus thermophiles* TTHB178 and *E*. *coli* RNase T, were identified as DNA repair–related exonucleases [33,38]. Both enzymes can remove deaminated bases like dI (also named hypoxanthine) and uracil in ssDNA with similar efficiencies as in digesting regular nitrogenous bases in vitro [33,38]. In addition, in vivo studies showed that TTHB178 and RNase T exhibited similar responses to the genotoxic stresses of H_2_O_2_ and UV irradiation [33,38].

Since TREX1 was shown to respond to such genotoxic stress by translocating to the nucleus [24], it would be of interest to characterize whether TREX1 can digest DNA substrates damaged by H_2_O_2_ and UV irradiation as TTHB178 and RNase T do. Therefore, we incubated TREX1 with ssDNA substrates that each contain a methylated base, a deaminated base, an oxidized base, or an abasic site at the 3′-terminal end (5′-GAGTCCTATAX-3′) and measured the activities of TREX1 in ssDNA degradation. The results presented in S2A Fig clearly showed that TREX1 exhibited similar activities in digesting ssDNAs containing a methylated base (O^4^-methylthymine [O^4^-mT] and O^6^-methylguanine [O^6^-mG]) or a deaminated base (uracil and hypoxanthine) as in digesting ssDNAs with regular bases (adenine). The ssDNA with an abasic site or an oxidized base (8-oxoguanine [8-oxoG]), though, was more resistant to TREX1. Therefore, the base preference of TREX1 to ssDNA substrates is similar to that of TTHB178 and RNase T in the DEDDh family.

RNase T showed in in vitro experiments that it can serve as a downstream exonuclease of Endonuclease V (Endo V)–mediated alternative base excision repair (AER) [33]. Endo V–mediated AER repairs DNA lesions originated from deamination of the adenine base (hypoxanthine), frame shift mutations, or replication errors [39–41]. The initiation step is making a nick at the 3′ side 1 base away from the damaged site. Expression of eukaryotic Endo V in DNA repair–deficient *E*. *coli* cells reduced the mutation frequency in the host, suggesting that the DNA repair function of Endo V may commonly display in *E*. *coli* and mammalian cells [40,41]. Therefore, TREX1 may also serve as a downstream exonuclease of Endo V, as RNase T does. In addition to DNA substrates, recent studies showed that both Endo V and TREX1 are also ribonucleases, as they exhibited considerable activities in digesting RNA substrates [28,32]. Despite the common ground at the level of enzyme activity, the involvement of TREX1 in this DNA repair pathway and its specific roles would require further investigation to establish.

**Fig 2:**
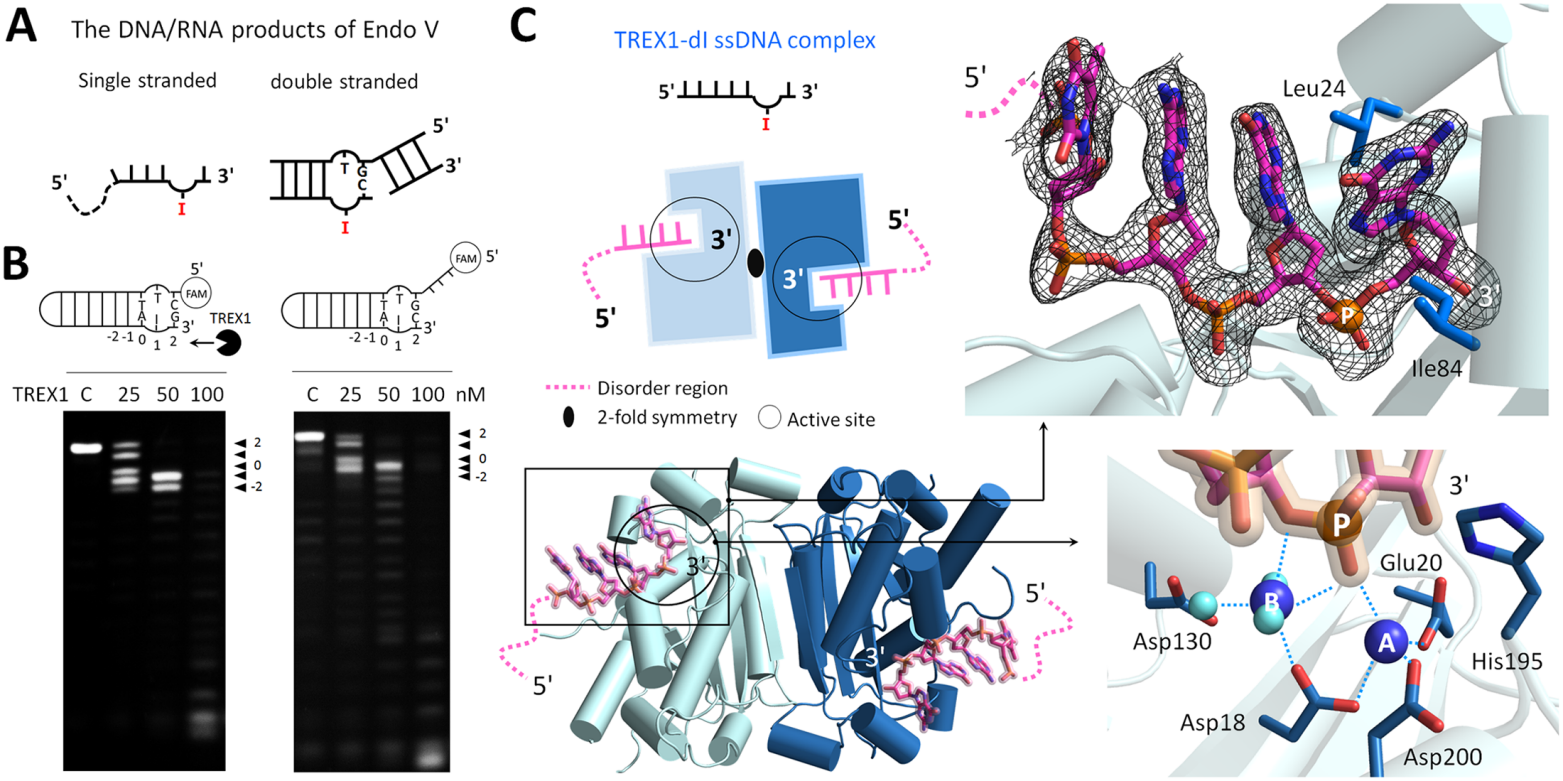
The TREX1-dI-ssDNA structure. (A) Schematic representation of the DNA/RNA products generated by Endo V. (B) The nuclease activities of TREX1 in digesting bubbled DNAs containing a hypoxanthine base (also named dI), including dI-bubbled DNA and dI-bubbled DNA with 5′-overhang. The concentration of all substrates was 0.5 μM. (C) An overview of the TREX1-dI-ssDNA structure. The upper panel shows the dI-ssDNA molecule in the TREX1-dI-ssDNA structure. The omitted electron density map (black) is contoured at 2.0 σ. Scissile phosphate, Mg^2+^, and water molecules are shown in orange, blue, and light blue balls, respectively. The hydrogen bonds between DNA, TREX1, water, and Mg^2+^ are marked with blue dotted lines. dI, deoxyinosine; Endo V, endonuclease V; Mg^2+^, magnesium ion; ssDNA, single-stranded DNA; TREX1, three prime repair exonuclease 1.

A prominent question, therefore, is whether TREX1 can process the structural DNAs generated by Endo V. In this regard, we designed and synthesized a bubbled dsDNA containing a dI with or without the 5′-overhang to mimic the end product of the enzyme (Fig 2A and 2B). This double-stranded substrate is referred to as dI-dsDNA. The Tris-borate-EDTA (TBE) gel electrophoresis was used to check the structure property of dI-dsDNA. With identical numbers of bases, the moving speed of dI-dsDNA in the gel was in between that of a Y-structural dsDNA and that of a regular dsDNA, indicating an intermediate, bubbled structure (S2B Fig). The results of our nuclease activity assay showed that TREX1 indeed broke the terminal base pairing and removed the last base pair near the penultimate hypoxanthine base at the 3′-end of dI-dsDNA (the 2 site labeled in Fig 2B). In addition, TREX1 also removed the penultimate hypoxanthine base and antepenultimate adenine base (the 1 and 0 sites labeled in Fig 2B). For the dI-dsDNA substrate with 5′-overhang, similar results were observed, indicating that the dangling 5′-ends did not affect TREX1 in processing the dsDNA.

In summary, our activity measurements established that TREX1 not only digested the dI in ssDNA; the enzyme can also extend into the duplex region and removed the last 3 bases at the 3′-end of a bubbled dI-dsDNA that mimics the product of Endo V. These results also highlighted that TREX1 is able to unwind the terminal base pairs in a DNA duplex to conduct digestion.

### TREX1 exhibits structure similarity in binding with dI-ssDNA and ssDNA

To provide the structural basis for TREX1 in complexing an ssDNA substrate containing dI, we determined the TREX1-dI-ssDNA structure. Fig 2C showed that the 3′-end of dI-ssDNA is inserted into the active site of TREX1 in each of the dimer in the asymmetric unit. The last nucleotide at the 3′-end is stacked by Leu24 and Ile84 (Fig 2C). Each active site contained 2 Mg^2+^, MgA and MgB. MgA coordinates with Asp18, Glu20, and Asp200 of TREX1 and was in contact with the phosphate oxygens of the scissile DNA strand. The His195 general base in the active site lacked the nucleophilic water that would bind MgA, a commonly observed configuration among the structures of DEDDh members [10,33,37]. MgB in the structure coordinated with 6 partners in the octahedral geometry, including Asp18, 2 phosphate oxygens in the scissile DNA stand, and 3 water molecules (Fig 2C). Both active sites of TREX1 in the asymmetric unit showed an active conformation as in the structures of other classical DEDDh exonucleases (S3A Fig).

In the TREX1-dI-ssDNA structure, the hypoxanthine base was designed to locate in the penultimate position at the 3′-end of dI-ssDNA as the products generated by Endo V (Fig 2A and 2C). The omitted electron density map of the hypoxanthine base in ssDNA fits very well to the three-dimensional structure (Fig 2C). Superposition of the structure of the TREX1-dI-ssDNA structure with that of TREX1 bound with a regular ssDNA showed that TREX1 bound both substrates with high structure similarity (S3B Fig). Therefore, the chemical modification into a hypoxanthine base at the penultimate position of the 3′-end did not affect the binding mode of TREX1 with ssDNA and its active site structure.

### The Leu24-Pro25-Ser26 cluster of TREX1 broke the terminal base pairings for processing dI-dsDNA

**Fig 3:**
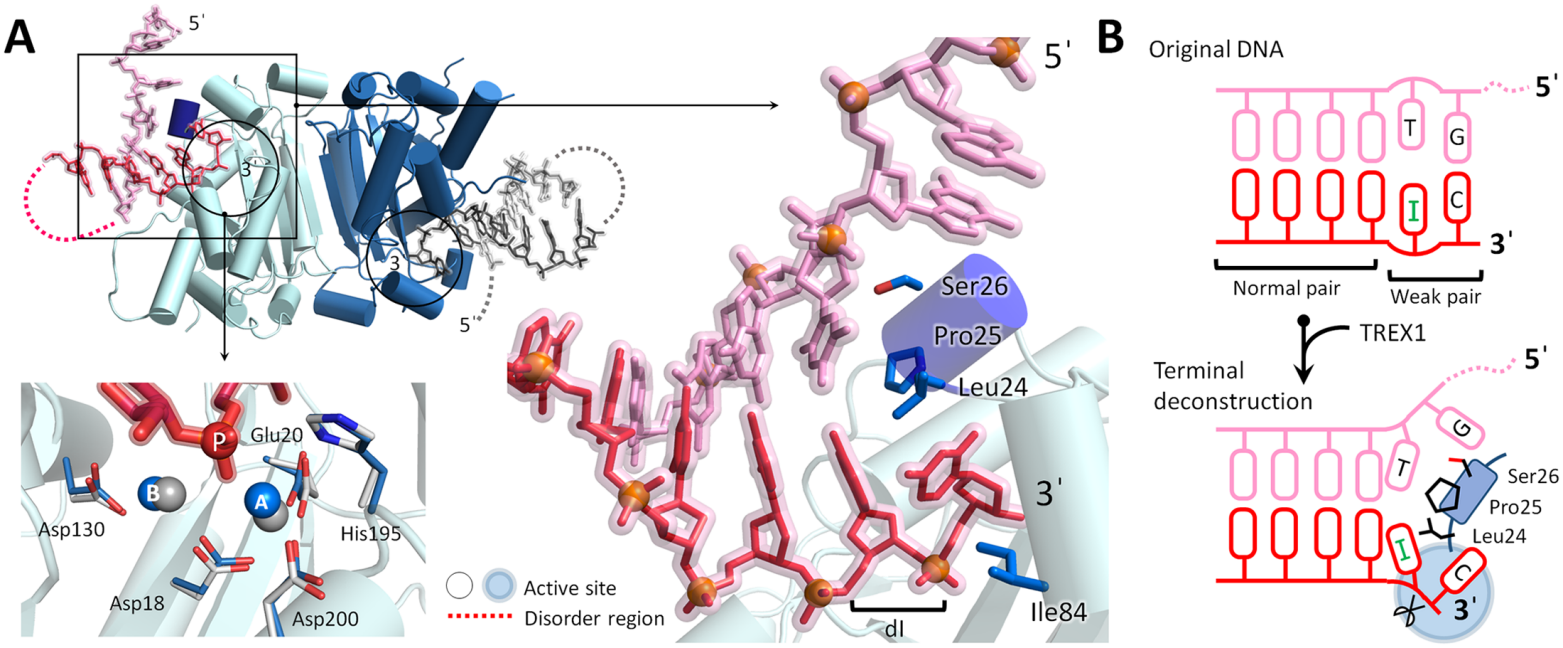
The TREX1-dI-T-dsDNA structure. (A) An overview of the TREX1-dI-T-dsDNA structure. The phosphate atoms of dsDNA in the TREX1-dI-T-dsDNA structure are shown as orange balls. The metal ions and active site residues in the TREX1-dI-ssDNA and TREX1-dI-T-dsDNA structures are colored in gray and blue, respectively. (B) Schematic representation for the Leu24-Pro25-Ser26 cluster wedging in the duplex end of dI-T-dsDNA. The scissile and nonscissile strands are colored in red and pink, respectively. dI, deoxyinosine; dsDNA, double-stranded DNA; ssDNA, single-stranded DNA; TREX1, three prime repair exonuclease 1.

In the TREX1-dI-T-dsDNA structure we solved, both protomers bound with a dI-dsDNA, as shown in Fig 3A. Both active sites of the enzyme dimer displayed the same active conformation as in the TREX1-dI-ssDNA structure. The omitted electron density maps in the double-strand region of both dI-dsDNA strands, though, were well defined for structure determination. In contrast, the electron densities of the 2 short 5′-overhang were not as continuous and weak, suggesting that the 3-nt-long 5′-overhangs were disordered (S4A Fig).

The structure of the TREX1-dI-T-dsDNA complex vividly illustrated the mode by which TREX1 broke the last base pairing in the duplex region. In particular, the Leu24-Pro25-Ser26 cluster acted as a wedge to chisel the duplex end formed by the last G-C pair and the dI-T wobble pair, and the DNA adopted a Y-like structure (Fig 3A and 3B). The complex of TREX1-dI-T-dsDNA hence provided a first structural basis for the ability of TREX1 in unwinding the duplex region of dsDNA. In addition to the Leu24-Pro25-Ser26 cluster, the structure indicated that the narrow pocket of the TREX1 active site was also involved in breaking the double-stranded structure as Leu24 and Ile84 stacked with the last nucleotide at the 3′-end (Fig 3A). Furthermore, the last nucleotide at the 3′-end of the substrate formed several hydrogen bonds with Glu20, Ala21, and Tyr129 in the narrow pocket (S4B Fig). These couplings to the 3′-end would thus facilitate unwinding of the terminal base pair. In summary, TREX1 exhibited the delicate machinery that the Leu24-Pro25-Ser26 cluster partnering with the narrow active site pocket to unwind the duplex end for digestion of dsDNA.

The mechanistic insights provided by the TREX1-dI-T-dsDNA complex structure were in line with the biochemical assay measurements presented earlier that TREX1 removed the 2 to −1 bases at the 3′-end of the bubbled dI-dsDNA (Fig 2B). Our results thus illustrated that the unique catalytic properties of TREX1 in digesting dsDNA and in processing the hypoxanthine base can serve to digest the product generated by Endo V. This finding represented an evidence that a DEDDh member like TREX1 and RNase T may act as a downstream exonuclease for Endo V–mediated AER in DNA repair.

### The structural basis for precise excision of 3′-overhang in structural DNAs by TREX1

**Fig 4:**
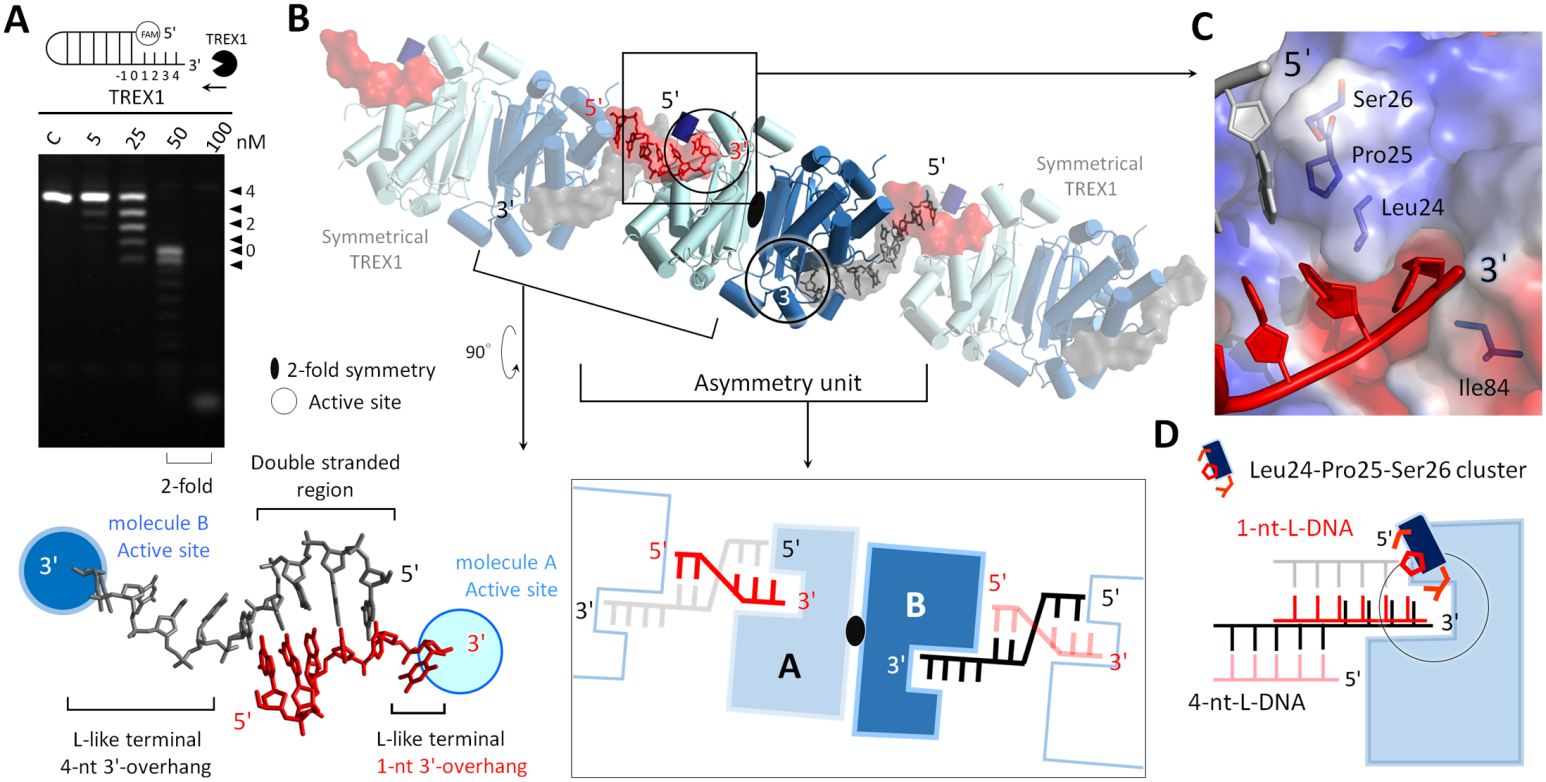
The TREX1-L-structural dsDNA structure. (A) The nuclease activities of TREX1 in digesting duplex DNAs with 4-nt-long 3′-overhang. (B) The asymmetric unit in the crystal contained 1 TREX1 dimer and 2 ssDNA molecules. The parts shown with a transparent mode depict the symmetry of TREX1 and DNA. The DNA duplex was formed by the ssDNAs bound to 2 TREX1 molecules in separate dimers. The 2 3′-ends of this duplex were 1 nt and 4 nt long and formed an L-like structure at each 3′-terminal, and they are referred to as 1-nt-L-DNA and 4-nt-L-DNA, respectively. (C) The 5′-ends of the duplex region in 1-nt-L-DNA were blocked by Leu24-Pro25-Ser26 cluster. (D) Schematic representation of the 2 modes for TREX1 binding with 1-nt-L-DNA and 4-nt-L-DNA. dsDNA, double-stranded DNA; ssDNA, single-stranded DNA; TREX1, three prime repair exonuclease 1.

**Fig 5:**
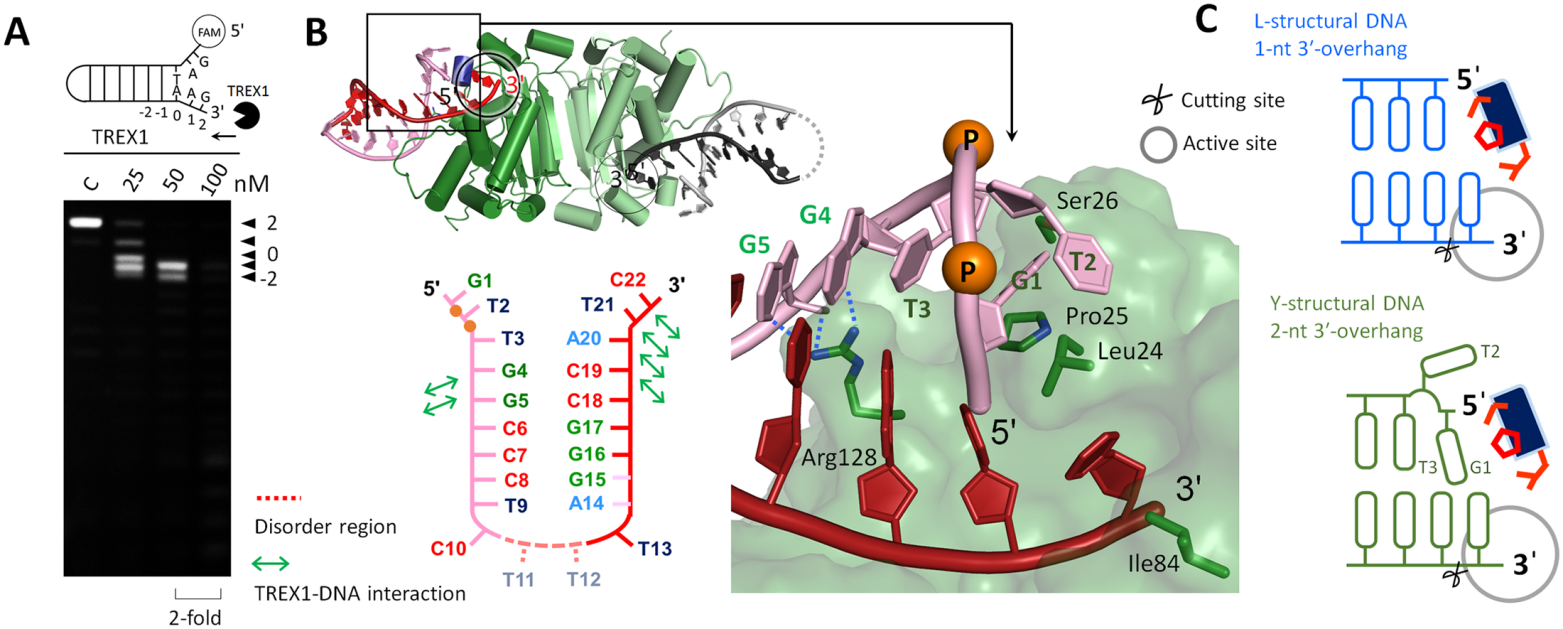
The TREX1-Y-structural dsDNA structure. (A) The nuclease activities of TREX1 in digesting Y-structural DNA. (B) An overview of the TREX1-Y-structural dsDNA structure. The last 2 phosphates in the 5′-end are displayed as orange balls and labeled as “P.” Arg128 interacted with the G4 and G5 bases via hydrogen bonding. (C) Schematic comparison of the TREX1-Y-structural dsDNA and the TREX1-L-structural dsDNA structures (1-nt-L-DNA). The cutting sites are labeled by scissors. dsDNA, double-stranded DNA; TREX1, three prime repair exonuclease 1.

Our activity measurements showed that TREX1 can remove the single-stranded regions in structural DNAs, and blunt-end duplexes are a main form of product of TREX1 (Figs 4A and 5A). To uncover the molecular origin of such precise removal of the 3′-overhang, we determined the structures of TREX1 complexed with the substrates that we devised to mimic the intermediates in UV-induced DNA repair. The structures of TREX1-L-structural dsDNA and TREX1-Y-structural dsDNA were solved at 1.7 Å and 2.0 Å resolution, respectively. The protein residues interacting with the DNA substrates in these two structures are shown in S5 Fig. The active sites in both structures also adopted the active form of conformation. Both structures contain regions of short 3′-overhang (1-nt- or 2-nt-long) to illustrate how the 3′-overhang was removed.

In the TREX1-L-structural dsDNA structure, 1 asymmetric unit contained 2 TREX1 monomers and 2 ssDNAs of 6 and 9 nt in length (Fig 4B). The 2 strands paired to their symmetric ssDNA in the crystal via GC-rich regions. The annealed DNA strands formed 2 duplexes of 1-nt- and 4-nt-long 3′-overhang, respectively. Each 3′-terminus of the DNAs displayed an L-like conformation, and for both duplexes, the 3′-overhang was inserted into the narrow active site of a TREX1 monomer. We designate molecule A as the TREX1 monomer that bound with the duplex DNA with a 1-nt-long 3′-overhang, 1-nt-L-DNA and molecule B as the TREX1 monomer that bound with the DNA duplex with a 4-nt-long 3′-overhang, 4-nt-L-DNA (Fig 4B).

Superposition of 1-nt-L-DNA and 4-nt-L-DNA revealed 2 distinct modes of binding duplex DNA for molecule A and molecule B (Fig 4D and S6A Fig). It can be seen clearly that the duplex segments of 1-nt-L-DNA and 4-nt-L-DNA sit at 2 different loci with respect to the protein. The Leu24-Pro25-Ser26 cluster of molecule A capped the 5′-end of 1-nt-L-DNA via contact with the last base of the nonscissile strand (Fig 4C). Leu24 and Ile84 in the narrow pocket of the active site in molecule A also stacked with the last nucleotide at the 3′-end of the scissile strand as in the TREX1-dI-T-dsDNA structure, indicating joint actions of the two structure motifs for removal of the 3′-overhang. Molecule A also formed 4 hydrogen bonds with the nonscissile strand of 1-nt-L-DNA and via Ser26, Arg128, and Lys160. In molecule B, however, it was a groove composed of Ala161, Leu162, Ala214, Gln217, and Trp218 on the other side of the Leu24-Pro25-Ser26 cluster that contacted with the 5′-end of 4-nt-L-DNA (S6B Fig). Moreover, molecule B did not form any hydrogen bonds or stacking interactions with the nonscissile strand of 4-nt-L-DNA.

Therefore, the TREX1-L-structural dsDNA structure showed that, in trimming a duplex DNA to generate duplexes with blunt ends, TREX1 can cap the nonscissile 5′-end via the Leu24-Pro25-Ser26 cluster and form hydrogen bonds and stacking interactions with the nonscissile strand. Such binding mode was neither observed in the case of molecule B bound with 4-nt-L-DNA (Fig 4D and S6B Fig) nor in earlier structures of TREX1 with substrates that contain a longer 3′-overhang [10].

The binding mode of Leu24-Pro25-Ser26 capping the nonscissile 5′-end was also observed in the TREX1-Y-structural dsDNA structure. In this case, the crystal also contained 2 TREX1 molecules in the asymmetry unit, and both bound with a Y-structural DNA of 2-nt-long 3′- and 5′-overhang. The sequence and structure of the substrate are shown in Fig 5B. The scissile strand of the Y-structural DNA was trapped by TREX1 via more than 10 amino acids, and the last nucleotide of the 3′-end was inserted into the active site and stacked by Leu24 and Ile84 in the narrow active site pocket, as observed in the structures of TREX1-dI-T-dsDNA and TREX1-L-structural dsDNA. The last base (G1) at the 5′-overhang of the substrate sits in the gap between TREX1 and the last base at the duplex region of Y-structural DNA and was flanked by the Leu24-Pro25-Ser26 cluster of TREX1 and the T3 base on the nonscissile strand (Fig 5B and 5C). With the nonpairing T2 base of the nonscissile strand flipped out, this binding mode is similar to that of molecule A bound with 1-nt-L-DNA in the TREX1-L-structural dsDNA structure (Fig 5C).

Therefore, both structures that contain duplex DNAs with a short 3′-overhang revealed the binding mode of Leu24-Pro25-Ser26 cluster in TREX1 capping the nonscissile 5′-end. Signature of this mode also highlighted the joint actions of the Leu24-Pro25-Ser26 cluster and the active site pocket in coupling to the 3′-end of the scissile strand, and specific interactions with the nonscissile strand were observed as well. These results provide the structural basis for duplex DNAs without a 3′-overhang being a major product form of TREX1 in activity assays (Figs 4A and 5A).

### The molecular basis and regulation mechanisms of TREX1 in dsDNA degradation

**Fig 6:**
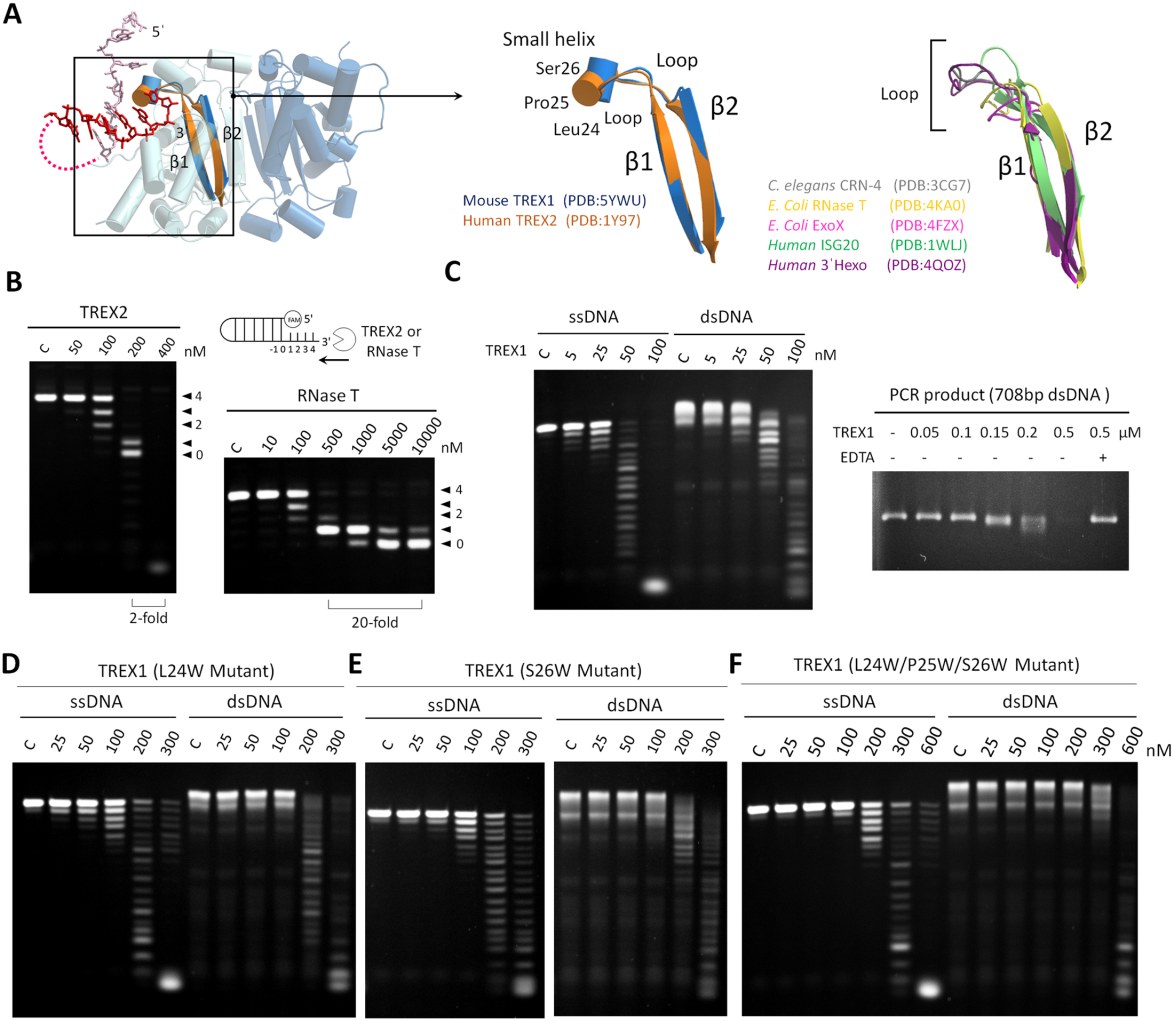
The terminal unwinding activity of TREX1 empowered by the Leu24-Pro25-Ser26 cluster. (A) Structure comparison of the loop region between the highly conserved β1 and β2 of TREX1, TREX2, and other classical DEDDh exonucleases. The PDB structures used in the structural alignment are mouse TREX1 (PDB accession code: 5YWU), human TREX2 (PDB accession code: 1Y97), *Caenorhabditis elegans* CRN-4 (PDB accession code: 3CG7), *E*. *coli* RNase T (PDB accession code: 4KA0), *E*. *coli* ExoX (PDB accession code: 4FZX), human ISG20 (PDB accession code: 31WLJ), and human 3′Hexo (PDB accession code: 4QOZ). In this region, only TREX1 and TREX2 contain a small helix that starts the Leu24-Pro25-Ser26 cluster. (B) The nuclease activities of TREX2 and RNase T in digesting duplex DNAs with 3′-overhang. The concentration of duplex DNA was 0.5 μM. TREX2 exhibited activities in digesting the duplex regions of DNA. RNase T at 500 nM only removed the 3′-overhang in duplex DNA and produced a duplex DNA with 1- or 0-nucleotide 3′-overhang. When the concentration of RNase T was increased by 20-fold to 10,000 nM, the double-strand structure of the substrates still persisted. (C) The nuclease activities of wild-type TREX1 in digesting ssDNA, dsDNA, and a PCR product (A linear 708 bp dsDNA). The concentration of ssDNA and dsDNA were 0.5 μM. The amount of the PCR product was 300 ng, and the concentration of EDTA was 5 mM. (D)(E)(F) The nuclease activities of 3 TREX1 mutants (L24W, S26W, and L24W/P25W/S26W) in digesting ssDNA and dsDNA substrates. dsDNA, double-stranded DNA; PDB, Protein Data Bank; ssDNA, single-stranded DNA; TREX1, three prime repair exonuclease 1.

The Leu24-Pro25-Ser26 cluster that wedged the nonscissile 5′-end in the TREX1-dI-T-dsDNA complex structure located at the N-terminal of a short helix of Pro25 to Ser27, and the helix was linked to the β strands of β1 and β2 by 2 loops. Although β1 and β2 are highly conserved, bioinformatics analysis indicated that the sequence and structure of the region in between exhibits significant variation in the DEDDh exonuclease family (Fig 6A and S7A Fig). Only TREX1 and TREX2 in the family, the corresponding region of Pro25 to Ser27, adopted a wedge-liked structure involving a small helix, whereas the corresponding region in other family members adopted a loop form of structure (Fig 6A). In targeting a single-stranded substrate, the X-ray structures of TREX1 resolved in this work (the TREX1-dI-ssDNA structure, Fig 2C) and those of RNase T [35] indicated that this region stacked the substrate with residues in the active site pocket in either a helical or loop form of secondary structure, and the nuclease activities of TREX1 and RNase T toward ssDNAs were similar.

In TREX1, the helical form of the Leu24-Pro25-Ser26 cluster in between β1 and β2 shaped a wedge-like structure, which can cap the 5′-end for producing blunt-end duplexes (the TREX1-L-structural dsDNA structure, Fig 4B and the TREX1-Y-structural dsDNA structure, Fig 5B) and even overcome the hindrance of duplex regions for processing dsDNAs (the TREX1-dI-T-dsDNA structure, Fig 3). Such unique catalytic powers compared to other members in the DEDDh family echoed the distinct helical structure form of the Leu24-Pro25-Ser26 cluster in TREX1 and the concomitant higher mechanical strength. With a loop form of structure in the corresponding region, the nuclease assay of *E*. *coli* RNase T did not reveal any activity toward dsDNA, even at an enzyme concentration as high as 10 μM under the same reaction conditions (Fig 6B and S7B Fig). The capability of digesting duplex structures was only observed for the closely related homolog TREX2 that also contained a wedge helix (Fig 6B and S7B Fig).

Indeed, over the incubation time of our nuclease activity assays, TREX1 was shown to fully consume ssDNA as well as dsDNA, albeit the enzyme concentration for the double-stranded substrate was higher (Fig 6C). To further examine the roles of Leu24-Pro25-Ser26 cluster in the activity of TREX1, we generated 4 single-site mutants (L24G, L24A, L24W and S26W) and 2 triple mutants (L24G/P25G/S26G and L24W/P25W/S26W) of the enzyme and measured the activities of these TREX1 mutants against ssDNA and dsDNA substrates. In comparison to the wild type, all mutants showed reduced activities toward both dsDNA and ssDNA substrates (Fig 6D, 6E and 6F and S7C, S7D and S7E Fig). Therefore, it can be inferred that perturbations to the Leu24-Pro25-Ser26 cluster due to these mutations affected not only the ability of stacking the last base at the 3′-end (with Ile84) to properly position the substrate in the active site for catalysis (Figs 2C, 3A, 4C and 5B); they also eliminated the power of breaking the terminal base pairing. Our mutagenesis and activity characterizations highlighted the importance of the Leu24-Pro25-Ser26 cluster in both terminal unwinding of dsDNA and 3′-ended nucleotide stacking for TREX1.

As the enzyme concentration was further raised to 500 nM, the measured activity for digesting the duplex regions was also augmented to accomplish full digestion of dsDNA as long as 708 bp (Fig 6C). At an enzyme concentration of 50 nM, on the other hand, TREX1 processed the ssDNA region of 3′-overhang in double-stranded substrates, leaving a blunt-end dsDNA (Figs 4A and 5A); the enzyme also trimmed the weaker pairing of a damaged base at a duplex terminal, as in the case of a nicked DNA generated by Endo V (Fig 2B). These results showed that the dsDNA degradation activity is concentration dependent, and under the in vivo scenario, other factors could also come to modulate the activity. For example, it is highly likely that the activity of TREX1 is coupled with partner proteins such as high mobility group box 2 (HMGB-2). HMGB-2 is also a component of ER-associated SET complex and is related to cytosolic nucleic acid–mediated innate immune responses [42–44]. The activity of HMGB-2 in bending the dsDNA structure potentially would generate distorted structures to facilitate dsDNA degradation by TREX1. Our preliminary results of activity measurements showed raised ability of TREX1 in digesting PCR products in the presence of mouse HMGB-2 (mHMGB-2) (S9 Fig). With mHMGB-2, TREX1 can fully digest PCR product at a lower enzyme concentration of 0.2 μM, evidencing the interplay between mHMGB-2 and TREX1 in modulating DNase activities.

## Discussion

### Structures of TREX1 with dsDNA that contained a short 3′-overhang revealed key mechanistic information of terminal excision and unwinding

**Fig 7:**
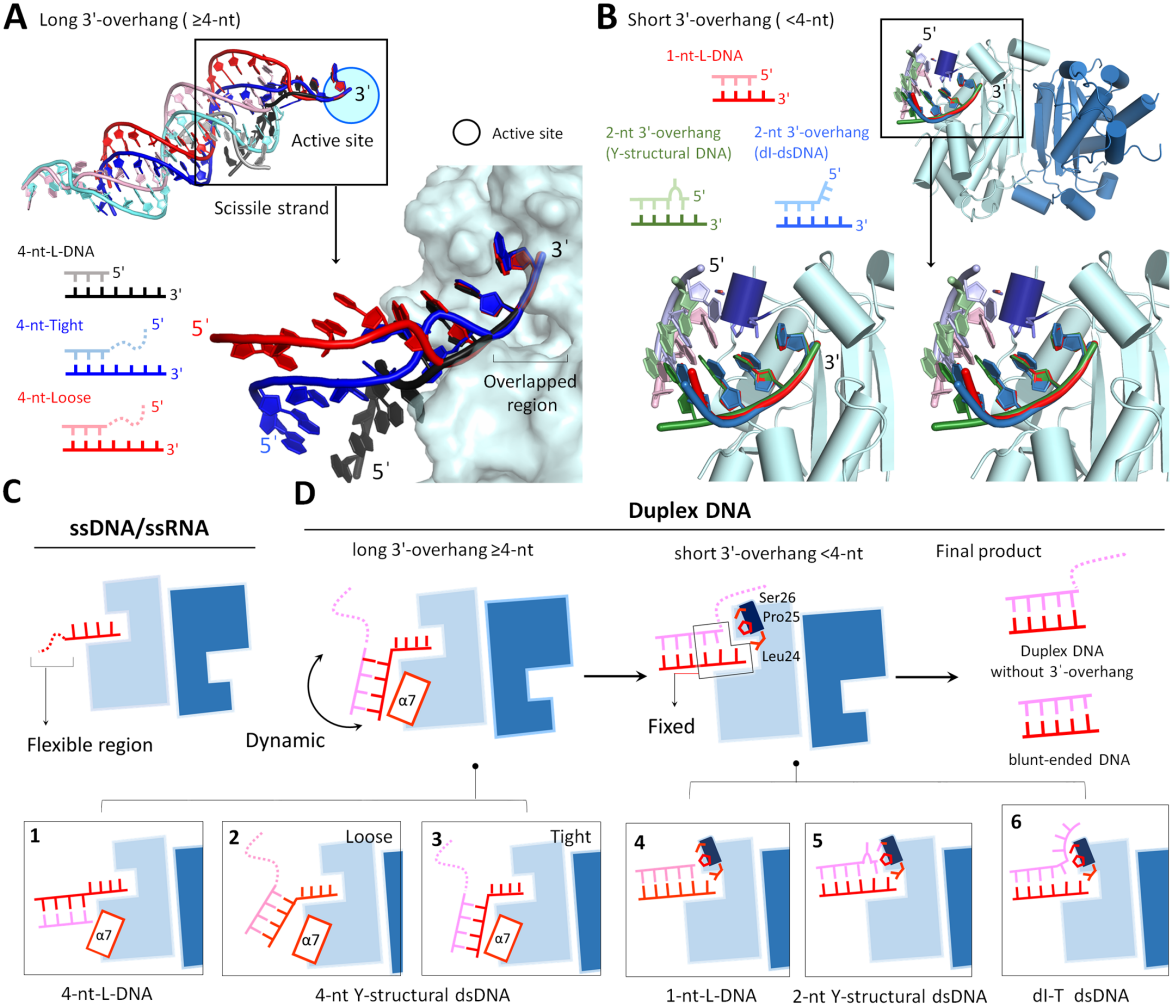
Structure comparison for the binding modes of TREX1 with various DNA substrates. (A) Superposition of the three structures of TREX1 in complexing with a duplex DNA with a long 3′-overhang (≥4-nt), including 4-nt-long 3′-overhang (4-nt-L-DNA) in the TREX1-L-structural dsDNA structure and the tight and loose conformations in the previous structures of TREX1-dsDNA complex (PDB accession code: 4YNQ). The colors and schematic diagrams of 3 duplex DNAs are displayed in the bottom panel. The right panel shows a close look for the difference between the scissile strands in the 3 duplex DNAs. The relative positions of the scissile strand of the duplex DNA in these 3 structures are different, only the positions of the last 2 nucleotides at the 3′-end of the scissile strand can fit well with each other. (B) Superposition of the three structures of TREX1 in complexing with a duplex DNA with a short 3′-overhang (<4-nt), including 1-nt-long 3′-overhang in the TREX1-L-structural dsDNA structure (1-nt-L-DNA), Y-structural DNA with 2-nt-long 3′-overhang in the TREX1-Y-structural dsDNA structure, and dI-containing dsDNA in the TREX1-dI-T-dsDNA structure. Comparison of these structures indicates the similarity of the binding regions between TREX1 and the 3′-end and 5′-end of the three types of dsDNA substrates. (C) Binding mode 1 is TREX1 with ssDNA substrates. (D) Binding mode 2 is TREX1 with duplexes of a long 3′-overhang, schematic representations in Boxes 1, 2, and 3. The PDB accession codes of these structures are 5YWT (4-nt-long L-structural dsDNA), 4YNQ (tight conformation), and 4YNQ (loose conformation). Binding mode 3 is TREX1 with a duplex of a short 3′-overhang, schematic representations in Boxes 4, 5, and 6. The PDB accession codes of these structures are 5YWT (1-nt-long L-structural dsDNA), 5YWS (2-nt-long Y-structural dsDNA), and 5YWU (dI-T-dsDNA). The scissile and nonscissile strands are colored in red and pink, respectively. α7 is the seventh α-helix (152–162 a.a). dI, deoxyinosine; dsDNA, double-stranded DNA; PDB, Protein Data Bank; ssDNA, single-stranded DNA; ssRNA, single-stranded RNA; TREX1, three prime repair exonuclease 1.

To date, the reported structures of duplex-bound TREX1 contained a substrate with a long 3′-overhang (≥4-nt-long) [10]. As a result, consensus features in such structures of binding were only observed for the last 2 nucleotides at the end of the 3′-overhang, while the farther-away regions of the scissile and nonscissile strands exhibited different directions and orientations (Fig 7A and S8A Fig). Therefore, using dsDNA substrates with a long 3′-overhang experienced significant difficulties in revealing the molecular mechanism by which TREX1 conducts overhang trimming and terminal unwinding.

On the contrary, a consistent mode of binding was observed in the structures resolved in this work that TREX1 complexed a dsDNA with a short 3′-overhang. The 3′-end of the scissile strand and the 5′-end of the nonscissile strand (Fig 7B) exhibited reproducible patterns in the TREX1-L-structural dsDNA, TREX1-Y-structural dsDNA, and TREX1-dI-T-dsDNA structures. For example, stacking between the nonscissile strand and the Leu24-Pro25-Ser26 cluster as well as the hydrogen bonding between Arg128 and the nonscissile strand (S4 and S5 Figs) are both prominent features that were commonly observed in these structures.

### Principles of TREX1 in binding and processing DNA substrates

Based on the 4 structures newly resolved in this work and literature data, we summarize the binding modes of TREX1 with various structural DNAs in Fig 7C and 7D. The first mode is for ssDNAs longer than 4 nt and, in this case, TREX1 bound to the last 4 nucleotides at the 3′-end with the 5′-end of the strand dangling. For damaged ssDNAs with hypoxanthine bases, we showed that TREX1 digested such substrates with a similar activity as degrading DNAs with normal nitrogenous bases. The ability of TREX1 in processing dI-ssDNA also pointed to a possible role in Endo V–mediated AER. TREX1, though, lacked the activity of cutting abasic site and 8-oxoG sites as the extra oxygen could hinder the enzyme to stack bases in the substrate. The resistance of 8-oxoG to TREX1 appears as an intrinsic design for transferring the signal of oxidative DNA damage by triggering stimulator of interferon genes complex (STING)–dependent immune sensing in cytoplasm [45]. Two members in DEDDh exonuclease family, *T*. *thermophiles* TTHB178 and *E*. *coli* RNase T, are homologous to TREX1 and demonstrated similar base preference, suggesting that these proteins perform similar functions in oxidative DNA damage signaling.

The second mode is for the class of duplex L- and Y-structural DNA substrates with a long 3′-overhang (≥4 nt), and most of the hydrogen bonds formed between TREX1 and the substrate were in the last 3 nucleotides at the 3′-end, i.e., in the 3′-overhang region. The nonscissile strand, on the other hand, did not land on consensus positions on the protein surface (Fig 7A and S8 Fig). Therefore, in binding DNA duplexes with a longer 3′-overhang, TREX1 more tightly coupled to the last 3 bases of the 3′-overhang region, and the rest of the substrate exhibited different conformations (Fig 7D).

For structural DNAs containing a short (<4 nt) 3′-overhang, the structures resolved in this work revealed the third class of TREX1-DNA binding. The Leu24-Pro25-Ser26 cluster in TREX1 was identified as a wedge to stack and interact with the 5′-end of the nonscissile strand, particularly at the last base in the double-stranded segment. Such specific couplings of capping the nonscissile 5′-end were not observed for TREX1 structures in binding with substrates that contained a longer 3′-overhang and provided a clear structural basis for the generation of blunt-end duplexes as the main product form after removal of the 3′-overhang (Fig 7D).

Our structure analysis also showed that the Leu24-Pro25-Ser26 cluster plays a critical role for TREX1 to break the terminal base pairing in dsDNAs. In the activity assays of TREX1 with duplex DNAs, minor products that lacked the last 1 or 2 nucleotides at 3′-end in the double-strand region were also observed (Figs 2B, 4A and 5A). For the structure with a wobble base pair shown in Fig 3, the last base pair was indeed separated for digestion. Together, these results indicate that the Leu24-Pro25-Ser26 cluster and other specific interactions enable TREX1 to break the base pairing around the nonscissile 5′-end and hence allow further nucleotide removal in the scissile strand. The terminal unwinding activity, in fact, allowed TREX1 to process the wobble-paired dsDNA generated by Endo V and to conduct dsDNA degradation as shown in Figs 2B and 6C.

Another key residue that was identified through the TREX1 structures resolved in this work is Arg128, which makes several contacts with the nonscissile strand in a duplex. As stated earlier, for all of the dsDNA substrates that we designed to have a short (<4 nt) 3′-overhang, the specific interactions of Arg128 are with the nonscissile strand. On the contrary, in the structures of TREX1 with a dsDNA containing a long 3′-overhang, Arg128 instead made contact with the single-stranded region of the scissile strand, and the mode of interactions is similar to that seen in the TREX1 structures with an ssDNA [10,31,46]. Mutation of the satellite residue Arg128 in TREX1 has been shown to result in an approximately 8-fold reduction in the activity of digesting dsDNA, but only about 2-fold of activity reduction was observed for ssDNA degradation [47]. This result highlighted the greater importance of Arg128 in dsDNA degradation. Our structures thus provide the necessary molecular details for understanding this result as the binding mode of TREX1 with a DNA duplex that contains a short 3′-overhang is different from that of binding an ssDNA. Since dsDNAs are primary substrates for immune silencing, our results also provide structure basis for the in vivo observation that Arg128 mutation is related to an autoimmune disease of SLE [20].

### On the roles of TREX1 in nuclear DNA repair and proofreading

A mystery of TREX1 in DNA repair is that the rate of spontaneous mutation in *TREX1*^*-/-*^ mice did not increase [18], despite the fact that genotoxic stress did lead to translocation of TREX1 to the nucleus and elevated expression levels of the enzyme [3,24]. A hypothesis that offers a potential explanation is participation of TREX1 in DNA repair processes like Endo V–mediated AER, and in this case, other pathways such as base excision repair (BER) for repairing DNAs with a hypoxanthine base would functionally overlap with those of TREX1 [48–50]. Although this scenario is subject to further studies to firmly establish, it is in line with the substrate compatibility of TREX1 with Endo V products, as observed in our activity characterization. As such, a single-gene knockout in either of the two overlapping pathways might not result in a significant increase in the rate of spontaneous mutation. An analogue example was that double mutations of DNA glycosylase and Endo V (*nfi*) were shown as necessary for an increased rate of spontaneous mutation to be observed, and transforming only intact gene 1 of 2 into the doubly mutated *E*. *coli* was sufficient to rescue the phenotype of a higher rate of spontaneous mutation [40,41].

Entanglement of multiple pathways would thus complicate the characterization of physiological impact for TREX1, and it is thus essential to consider that this enzyme is likely coupled to other partners in DNA repair–related processes. For example, TREX1 is also expected to respond to UV light–induced DNA stress since translocation of the enzyme was observed upon exposure to UV illumination [24]. The consequent repairing processes include removal of L- or Y-structural dsDNA in the replication restart pathway, gap-filling homologous recombination, and RecA-dependent homologous recombination that the TREX1 homolog *E*. *coli* RNase T is also known to perform [33]. In this regard, the activity of TREX1 in trimming Y-structural dsDNA probably overlaps with that of the endonuclease Mus81-Mms4 (Slx2-Slx3), which also targets similar structures [51,52]. Along a similar line, the proofreading function of TREX1 in BER would overlap with that of Ape1, which bears a 3′–5′ exonuclease activity and interacts with DNA polymerase β in editing mismatched nucleotides [53–55]. Furthermore, TREX1 may also involve in other DNA repair pathways, such as by interacting with and regulating PARP-1, a DNA repair enzyme for which the molecular details await further studies to resolve [25]. The overlapping model thus provides a rationale for the unaffected rates of spontaneous mutation in TREX1 knockout mice, and for the proficiency of double-strand break (DSB) repair and BER in TREX1-deficient fibroblasts [5,18].

It is important to highlight that the DNA repair functions require TREX1 to be present in the nucleus. Translocation of TREX1 to the nucleus was observed during GzmA-mediated apoptosis or under genotoxic stresses in several works via immunoprecipitation and/or immunofluorescence microscopy [3,23,24]. TREX1 was also found in the nucleus and was shown to interact with a well-known DNA repair enzyme, PARP-1, via western blotting and coimmunoprecipitation [25]. However, TREX1 translocation to the nucleus was not observed in the immunofluorescence microscopy data of a recent work [5], suggesting the need of further studies to characterize the DNA repair function of TREX1.

TREX1 provides the major 3′–5′ exonuclease activity in mammalian cells [1,2] as numerous in vivo studies showed that TREX1 broadly displayed important roles in immune silencing [3,21], genotoxicity responses [3,24], apoptotic DNA degradation in dying cells [26], and chromosomal fragmentation during telomere crisis [27]. In this work, we aim to provide the structure basis for the molecular origin of TREX1 that enables such diverse activities toward a wide range of nucleic acid substrates, including ssDNA, dsDNA, DNA/RNA duplexes, and structural DNAs. The endeavor of crystallizing TREX1 with various duplex DNA substrates with a short 3′-overhang revealed the structure details for the unique catalytic powers of TREX1. We identified that the Leu24-Pro25-Ser26 cluster at the N-terminal of a short helix can cap the nonscissile 5′-end for trimming the 3′-overhang for producing a blunt-end duplex and can wedge into the duplex end to unwind the terminal base pairing in a dsDNA. These results also provide sophisticated molecular pictures for rationalizing the cellular functions of TREX1 in DNA repair, DNA proofreading, and immune silencing.

## Materials and methods

### Protein expression and purification

The *trex1* gene from *Mus musculus*, with a length of 1–242 amino acids, was subcloned into BamHI/XhoI site in plasmid pET28a. The *trex2* gene from *M*. *musculus* was subcloned into NdeI/XhoI site in plasmid pET28a. The expression vector was transformed into *E*. *coli* BL21-CodonPlus(DE3)-RIPL or Rosetta2(DE3)pLysS strain (Stratagene, United States) cultured in LB medium supplemented with 50 μg/mL Kanamycin. Cells were grown to an OD600 of 0.5–0.6 and induced by 1 mM IPTG at 18 °C for 20 h. The harvested cells were disrupted by sonication in 50 mM Tris-HCl (pH 7.5) containing 300 mM NaCl for 20 min. TREX1 and TREX2 were further purified by Ni-NTA resin affinity column (QIAGEN), HiTrap Heparin (GE Healthcare), and a Superdex 200 gel filtration column (GE Healthcare). The purified proteins in 50 mM Tris-HCl, pH 7.0 and 300 mM NaCl were concentrated to suitable concentrations and stored at −20 °C until use. All of the TREX1 mutants were generated by QuickChange site-directed mutagenesis kits (Stratagene) and purified by the same procedures as for wild-type TREX1. His-tagged TREX1 were treated with thrombin to generate non-His-tagged TREX1 for crystallization experiments. RNase T was purified as previous described [33].

### Nuclease activity assays

The sequences of DNA substrates are listed in S2 Table. DNA substrates were synthesized by BEX Co., Tokyo, Japan, or MDBio, Inc., Taiwan. Substrates were labeled at the 5′-end with γ-^32^P or FAM. The γ-^32^P was labeled at the 5′-end of substrate DNA by T4 polynucleotide kinase (New England Biolabs) and then purified by a Microspin G-25 column (GE Healthcare) to remove the nonincorporated nucleotides. Labeled substrates (0.5 μM) were then incubated with the exonuclease mixture in 120 mM NaCl and 20 mM Tris-HCl at pH 7.0 and 37 °C. The reaction was stopped by adding DNA-loading dye at 95 °C for 5 min. The digestion patterns were resolved on 20% denaturing polyacrylamide gels and visualized by autoradiography (Fujifilm, FLA-5000) or UV light. When the substrate was a PCR product, we added protease K into the reaction mixture at 37 °C for 1 h to stop the reaction. The DNA digestion patterns were resolved on 1% agarose gel and visualized by UV light.

### Crystallization and crystal structural determination

His-tagged or non-His-tagged TREX1 (15–25 mg/mL) in 300 mM NaCl and 50 mM Tris-HCl, pH 7.0 were mixed with different DNA substrates in the molar ratio of 1:1.5. Detailed information regarding DNA sequences and crystallization conditions of the 4 structures are given in S1 Table. All crystals were cryoprotected by Paraton-N (Hampton Research, USA) for data collection at BL13C1, BL13B1, and BL15A1 in NSRRC, Taiwan, or at the BL44XU beamline at SPring-8, Japan. All diffraction data were processed by HKL2000, and diffraction statistics are listed in Fig 1. Structures were solved by the molecular replacement method and using the crystal structure of *M*. *musculus* TREX1 (PDB: 3MXM) as the search model by MOLREP of CCP4 [56]. The models were built by Coot [57] and refined by Phenix [58]. Diffraction structure factors and structural coordinates have been deposited in the RCSB PDB with the PDB ID code of 5YWV for the TREX1-dI ssDNA complex, 5YWU for the TREX1-dI-T-dsDNA complex, 5YWT for the TREX1-L-structural dsDNA complex, and 5YWS for the TREX1-Y-structural dsDNA complex.

## Supporting information

S1 Table Crystallization conditions of TREX1-structural DNA complexes. TREX1, three prime repair exonuclease 1.
(DOCX)

S2 Table Substrates for biochemical studies. (DOCX)

S1 Fig Nuclease activity of TREX1 under various crystallization conditions. (A) 20 nM TREX1 was incubated with 0.5 μM 20 nt ssDNA under various conditions: 50% reaction buffer (1 mM MgCl_2_, 20 mM Tris-HCl pH 7.0, and 120 mM NaCl) and 50% crystallization buffers. The activity of TREX1 was inhibited or decreased under the crystallization conditions of TREX1-dI-ssDNA complex, TREX1-dI-T-dsDNA complex, and TREX1-Y-structural dsDNA complex. The activity of TREX1 was enhanced under the crystallization conditions of TREX1-L-structural dsDNA complex. (B) Denatured gel analysis for the DNA in the crystal of TREX1-dI-T-dsDNA complex. The crystal was dissolved and labeled at the 5′-end with γ-^32^P by a standard protocol. The band of the DNA from the crystal is located at the same position as the input DNA (control), suggesting that the DNA is not cleaved in the crystal. dI, dioxyinosine; dI-ssDNA, dI-T-dsDNA, dsDNA with a scissile strand containing a dI; ssDNA containing a dI; dsDNA, double-stranded DNA; ssDNA, single-stranded DNA; TREX1, three prime repair exonuclease 1.
(TIF)

S2 Fig Activity of TREX1 on DNA substrates with damaged bases. (A) For ssDNA substrates with different nucleotides in the 3′-end, including adenine, O^4^-mT, O^6^-mG, abasic site, 8-oxoG, uracil, and hypoxanthine. The 5′-ends of all of the substrates are labeled with γ-^32^P. The ssDNA with an abasic site is resistant to TREX1 even at concentrations of 200 nM. DNA with the 3′-terminal-oxidized base 8-oxoG also show a lower activity of TREX1 degradation. The most 8-oxoG–contained ssDNA is not degraded at 50 nM. (B) Substrate mobility assay of a hypoxanthine base containing bubble DNA (dI-dsDNA, Sample 1), ideally paired duplex DNA (sample 2), and Y-structural DNA (Sample 3). Three different DNAs with the same number of nucleotides were analyzed by 12% native TBE gel. Lanes 1 and 4 show sample 1, and lanes 2 and 3 indicate samples 2 and 3. The moving speed of dI-dsDNA is between samples 2 and 3, suggesting that the native structure of dI-dsDNA is different from the ideally paired duplex DNA and Y-structural DNA. 8-oxoG, 8-oxoguanine; dI, dioxyinosine; dsDNA, double-stranded DNA; O^4^-mT, O^4^-methylthymine; O^6^-mG, O^6^-methylguanine; ssDNA, single-stranded DNA; TBE, Tris-borate-EDTA; TREX1, three prime repair exonuclease 1.
(TIF)

S3 Fig Structural comparisons of the TREX1-dI-ssDNA and TREX1-ssDNA complexes. (A) Structural comparison of the active sites in TREX1-dI-ssDNA complex, TREX1-ssDNA complex (PDB Accession Code: 2OA8), and *ε* subunit of the DNA polymerase (PDB Accession Code: 1J53). (B) Structural comparison of the ssDNA in TREX1-dI-ssDNA and TREX1-ssDNA complex (PDB Accession Code: 2OA8). dI, dioxyinosine; dI-ssDNA, ssDNA containing a dI; PDB, Protein Data Bank; single-stranded DNA; TREX1, three prime repair exonuclease 1.
(TIF)

S4 Fig The interaction map between TREX1 and dI-dsDNA in the TREX1-dI-T-dsDNA complex. (A) The omitted electron density map (*F*o − *F*c, 1.0 σ) of 2 dI-dsDNAs in the TREX1-dI-T-dsDNA complex. The DNA with dotted lines indicates the disordered DNA regions in the crystal structure. (B) The upper right panel shows the schematic and overall structure of the TREX1-dI-T-dsDNA complex. The right bottom panel shows the surface of the Leu24-Pro25-Ser26 cluster. The left panel displays the schematic of the interactions between TREX1 and dI-containing dsDNA. dI, dioxyinosine; dI-T-dsDNA, dsDNA with a scissile strand containing a dI; dsDNA, double-stranded DNA; TREX1, three prime repair exonuclease 1.
(TIF)

S5 Fig Schematic of the interactions between TREX1 and structural DNAs. (A) For the structure of the TREX1-L-structural dsDNA complex. (B) For the structure of the TREX1-Y-structural dsDNA complex. dsDNA, double-stranded DNA; TREX1, three prime repair exonuclease 1.
(TIF)

S6 Fig Structures of the two TREX1 monomers (molecules A and B) with 1-nt-L-DNA and 4-nt-L-DNA. (A) Schematic of the two binding modes for TREX1 with 1-nt-L-DNA and 4-nt-L-DNA. Two 5′-ends of duplex DNA are blocked by different residues of 2 TREX1 protomers. Left and right panels show the blocking regions of TREX1 on 1 and 4 nt 3′-overhang, respectively. (B) Ala161, Leu162, Ala214, Gln217, and Trp218 in molecule B are in contact with the 5′-end of 4 nt L-DNA. However, no hydrogen bonds are formed in this region. TREX1, three prime repair exonuclease 1.
(TIF)

S7 Fig Sequence alignment and catalytic property assays of TREX1, TREX2, and RNase T. (A) Sequence alignment of TREX1 and other DEDDh exonucleases. The loop and helix regions between β-strand 1 (β1, 12–22 a.a.) and β-strand 2 (β2, 31–40 a.a.) are highlighted in blue and red, respectively. (B) Nuclease activities of TREX2 and RNase T on digesting Y-structural DNA. The structural restriction from a double-stranded structure at RNase T is stronger than that at TREX1 and TREX2. (C) (D) (E) Nuclease activities of 3 TREX1 mutants (L24A, L24G, and L24G/P25G/S26G) on digesting ssDNA and dsDNA. dsDNA, double-stranded DNA; ssDNA, single-stranded DNA; TREX1, three prime repair exonuclease 1; TREX2, three prime repair exonuclease 2.
(TIF)

S8 Fig The structural comparison of TREX1 in binding to duplex DNA with long 3′-overhang. (A) Superposition of the three structures of TREX1 in complex with duplex DNA with long 3′-overhang (≥4-nt), including 4-nt-long 3′-overhang (4 nt-L-DNA) in the TREX1-L-structural dsDNA complex and tight and loose conformations in the previous TREX1-dsDNA complex (PDB accession code: 4YNQ). The colors and schematic diagrams of 3 duplex DNAs are displayed in the top panel. The nonscissile strands of 3 duplex DNAs are in contact with TREX1 in different loci. For clarity, only partial nucleotides at the 5′-end of the nonscissile strands are shown. dsDNA, double-stranded DNA; PDB, Protein Data Bank; TREX1, three prime repair exonuclease 1.
(TIF)

S9 Fig DNA digesting assays of mTREX1 and mHMGB-2. (A) SDS-PAGE analysis of purified mHMGB-2 (1–210 a.a.). (B) Assays of TREX1 DNase activity in the presence or absence of mHMGB-2. A linear 708-bp dsDNA (300 ng) was incubated with mTREX1 and mHMGB-2, and the DNA digests were analyzed by gel electrophoresis. TREX1 cleaved linear dsDNA more efficiently in the presence of mHMGB-2 (by comparing with lane 5 and 8). dsDNA, double-stranded DNA; mHMGB-2, mouse HMGB-2; TREX1, three prime repair exonuclease 1.
(TIF)

## Abbreviations

8-oxoG: 8-oxoguanine
AER: alternative base excision repair
AGS: Aicardi-Goutières syndrome
BER: base excision repair
dI: deoxyinosine
dI-ssDNA: ssDNA containing a dI
dI-T-dsDNA: dsDNA with a scissile strand containing a dI
DSB: double-strand break
dsDNA: double-stranded DNA
Endo V: endonuclease V
ER: endoplasmic reticulum
FCL: familial chilblain lupus
GzmA: granzyme A
HIV-1: type 1 human immunodeficiency retrovirus
HMGB-2: high mobility group box 2
Mg^2+^: magnesium ion
mHMGB-2: mouse HMGB-2
NM23-H1: nonmetastatic protein 23 homolog 1
O^4^-mT: O^4^-methylthymine
O^6^-mG: O^6^-methylguanine
PARP-1: Poly [ADP-ribose] polymerase 1
Pfam: protein families
SET: patient SE translocation protein
SLE: systemic lupus erythematosus
ssDNA: single-stranded DNA
ssRNA: single-stranded RNA
STING: stimulator of interferon genes complex
TBE: Tris-borate-EDTA
TREX1: three prime repair exonuclease 1
