# Supplementary material for: Structural basis for overhang excision and terminal unwinding of DNA duplexes by TREX1
Source: PLoS Biol. 2018 May 7;16(5):e2005653. doi: 10.1371/journal.pbio.2005653 (PMC5957452; doi:10.1371/journal.pbio.2005653)
Supplement: S1 Table — TREX1, three prime repair exonuclease 1. (DOCX) [file pbio.2005653.s001.docx]

| **TREX1-dI-ssDNA complex** | | |
| --- | --- | --- |
| Protein : non-His-tagged TREX1  Input DNA:5′- TTATAIG -3′  Time of growth: 2 weeks  Condition: 0.1 M MES monohydrate pH 6.0,  20% w/v Polyethylene glycol monomethyl ether 2,000  Activity of TREX1: Reduced | Input DNA | DNA in the structure^a^ |
|  | **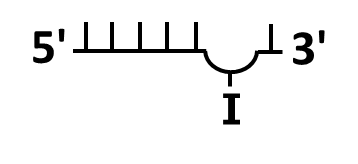** | **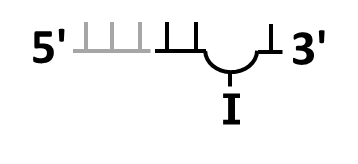** |
| **TREX1-dI-T-dsDNA complex** | | |
| Protein : non-His-tagged TREX1  Input DNA : 5′- AAAGTGGCCCTCTTTAGGGCCIC -3′  Time of growth: 3 to 9 weeks  Condition: 0.2 M Potassium citrate tribasic monohydrate,  pH 8.3, 20 % w/v Polyethylene glycol 3,350  Activity of TREX1: Inhibited | Input DNA | DNA in the structure^a^ |
|  | **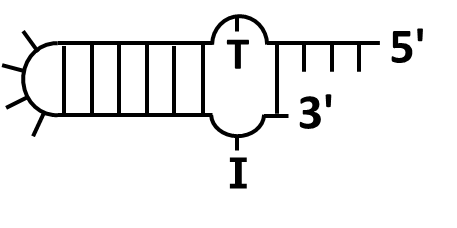** | **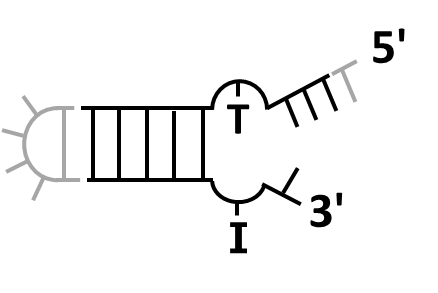** |
| **TREX1-L-structural dsDNA complex** | | |
| Protein : His-tagged TREX1  Input DNA : 5′- GGCCCTCTTTAGGGCCTTC -3′  Time of growth: 2-3 weeks  Condition: 0.1 M BICINE pH 8.5,  20% w/v Polyethylene glycol 10,000  Activity of TREX1: Enhanced | Input DNA | DNA in the structure^a, b^ |
|  | **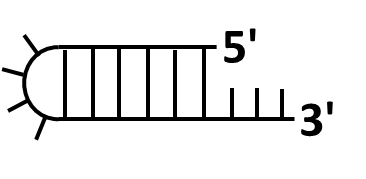** | **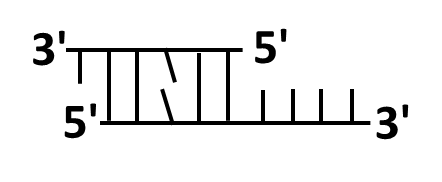** |
| **TREX1-Y-structural dsDNA complex** | | |
| Protein : His-tagged TREX1  Input DNA : 5′- GTTGGCCCTCTTTAGGGCCATC -3′  Time of growth: 2-6 weeks  Condition: 0.1 M Sodium acetate trihydrate pH 4.5,  30% v/v Polyethylene glycol 300  Activity of TREX1: Inhibited | Input DNA | DNA in the structure^a^ |
|  | **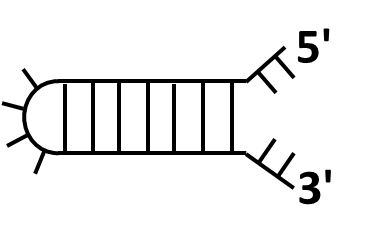** | **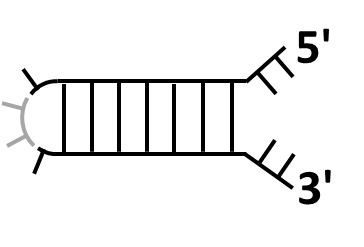** |

**S1 Table.** **Crystallization conditions of TREX1-structural DNA complexes**

^a^ DNAs displayed in gray are the disordered regions in the structures.

^b^ In the crystal of the TREX1-L-structural dsDNA complex, the input stem loop DNA was exonucleolytically digested by TREX1 into two small ssDNA fragments, including ssDNA with lengths of 6 and 9 nt (6 nt ssDNA: 5′-GGCCCT-3′; 9 nt ssDNA: 5′-GGCCCTCTT-3′). Two ssDNAs are annealed together to form a duplex DNA with 1-nt- and 4-nt-long 3′-overhangs. Each terminus displays an L-shape conformation.
