# Supplementary material for: Structural basis for overhang excision and terminal unwinding of DNA duplexes by TREX1
Source: PLoS Biol. 2018 May 7;16(5):e2005653. doi: 10.1371/journal.pbio.2005653 (PMC5957452; doi:10.1371/journal.pbio.2005653)
Supplement: S2 Table — (DOCX) [file pbio.2005653.s002.docx]

**S2 Table. Substrates for biochemical studies**

| **Substrate** | **Sequence** |
| --- | --- |
| ssDNA 20 nt | 5′- ACTGGACAAATACTCCGAGG -3′ |
| dsDNA 20 nt | 5′- ACTGGACAAATICTCCGAGG -3′  3′- TGACCTGTTTATGAGGCTCC -5′ |
| Stem-loop DNA with 3′-overhang (4 nt) | 5′- GGCCCTCTTTAGGGCCTTGG -3′ |
| Y-structural DNA (3′- and 5′-overhang : 4 nt) | 5′- TTAAGGCCCTCTTTAGGGCCAAGG -3′ |
| Y-structural DNA (3′- and 5′-overhang : 2 nt) | 5′- GATGGCCCTCTTTAGGGCCAAG -3′ |
| dI-bubbled DNA | 5′- CTTGGCCCTCTTTAGGGCCAIG -3′ |
| Perfect paired Stem-loop DNA | 5′- CTTGGCCCTCTTTAGGGCCAAG -3′ |
| dI-bubbled DNA with 5′-overhang | 5′- AAAGTTGCCCTCTTTAGGGCAIC -3′ |
| Single stranded DNA 11mer  with damaged DNA bases | 5′- AATCTTACAA **X** -3′  **X** : Adenine, O^4^-methylthymine (O^4^-mT), O^6^-methylguanine (O^6^-mG), 8-oxoguanine (8-oxo), abasic site, uracil or hypoxanthine (deoxyinosine). |

***** The underlined regions are the paired regions or stem regions of DNA substrates.
