# Supplementary figures and images for: Structural basis for overhang excision and terminal unwinding of DNA duplexes by TREX1
Source: PLoS Biol. 2018 May 7;16(5):e2005653. doi: 10.1371/journal.pbio.2005653 (PMC5957452; doi:10.1371/journal.pbio.2005653)

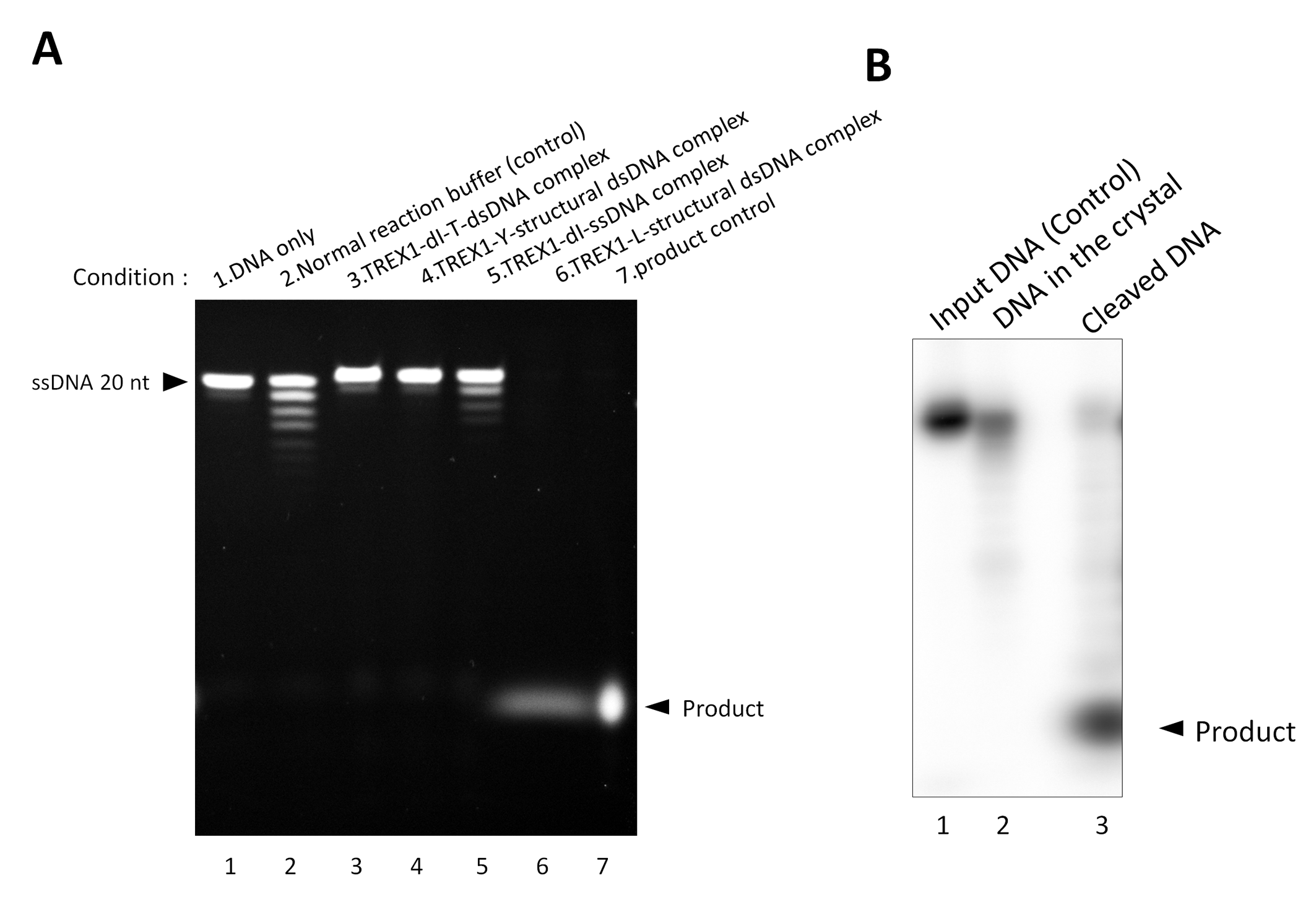

Supplement: S1 Fig — (A) 20 nM TREX1 was incubated with 0.5 μM 20 nt ssDNA under various conditions: 50% reaction buffer (1 mM MgCl2, 20 mM Tris-HCl pH 7.0, and 120 mM NaCl) and 50% crystallization buffers. The activity of TREX1 was inhibited or decreased under the crystallization conditions of TREX1-dI-ssDNA complex, TREX1-dI-T-dsDNA complex, and TREX1-Y-structural dsDNA complex. The activity of TREX1 was enhanced under the crystallization conditions of TREX1-L-structural dsDNA complex. (B) Denatured gel analysis for the DNA in the crystal of TREX1-dI-T-dsDNA complex. The crystal was dissolved and labeled at the 5′-end with γ-32P by a standard protocol. The band of the DNA from the crystal is located at the same position as the input DNA (control), suggesting that the DNA is not cleaved in the crystal. dI, dioxyinosine; dI-ssDNA, dI-T-dsDNA, dsDNA with a scissile strand containing a dI; ssDNA containing a dI; dsDNA, double-stranded DNA; ssDNA, single-stranded DNA; TREX1, three prime repair exonuclease 1. (TIF) [file pbio.2005653.s003.tif]

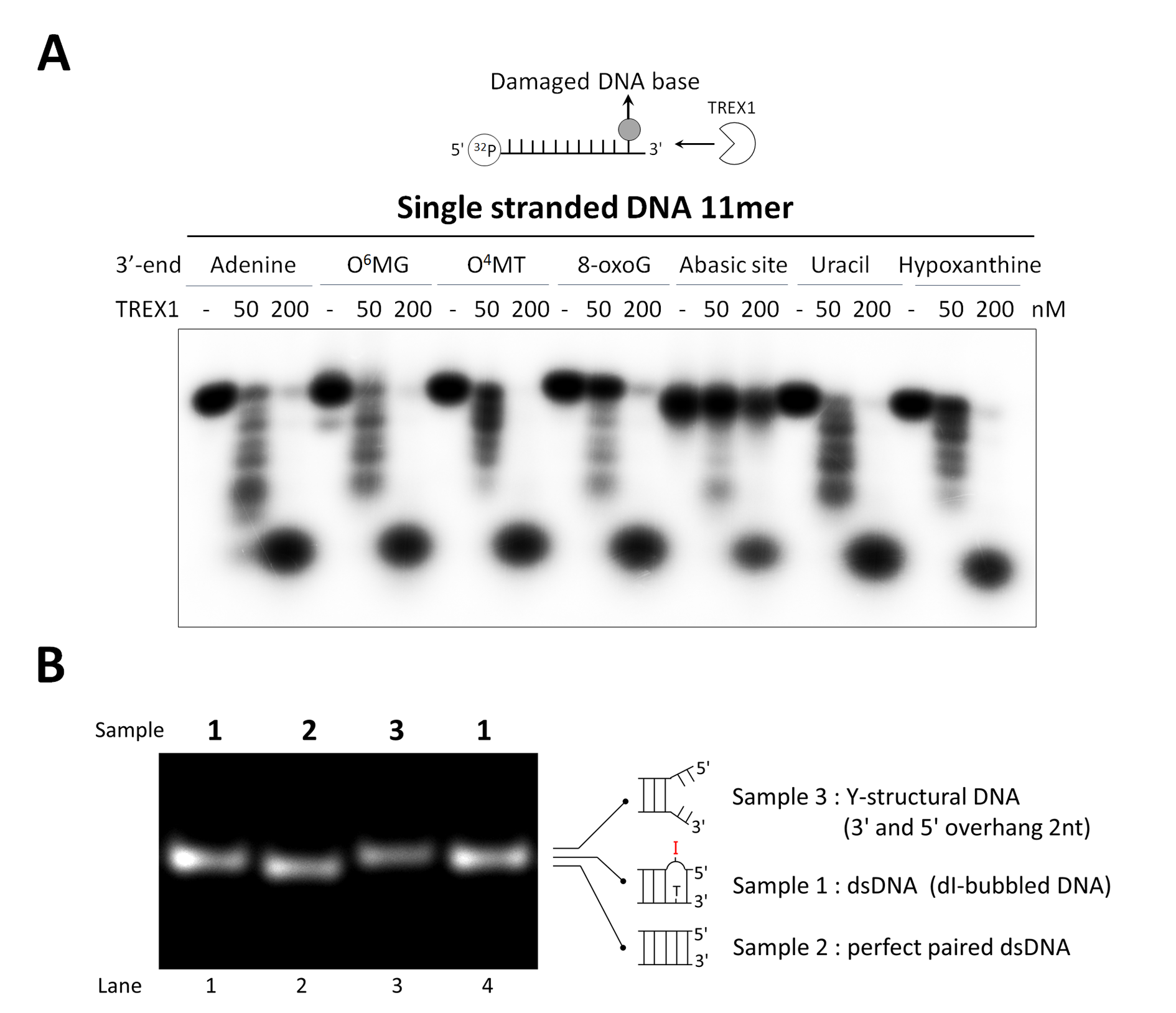

Supplement: S2 Fig — (A) For ssDNA substrates with different nucleotides in the 3′-end, including adenine, O4-mT, O6-mG, abasic site, 8-oxoG, uracil, and hypoxanthine. The 5′-ends of all of the substrates are labeled with γ-32P. The ssDNA with an abasic site is resistant to TREX1 even at concentrations of 200 nM. DNA with the 3′-terminal-oxidized base 8-oxoG also show a lower activity of TREX1 degradation. The most 8-oxoG–contained ssDNA is not degraded at 50 nM. (B) Substrate mobility assay of a hypoxanthine base containing bubble DNA (dI-dsDNA, Sample 1), ideally paired duplex DNA (sample 2), and Y-structural DNA (Sample 3). Three different DNAs with the same number of nucleotides were analyzed by 12% native TBE gel. Lanes 1 and 4 show sample 1, and lanes 2 and 3 indicate samples 2 and 3. The moving speed of dI-dsDNA is between samples 2 and 3, suggesting that the native structure of dI-dsDNA is different from the ideally paired duplex DNA and Y-structural DNA. 8-oxoG, 8-oxoguanine; dI, dioxyinosine; dsDNA, double-stranded DNA; O4-mT, O4-methylthymine; O6-mG, O6-methylguanine; ssDNA, single-stranded DNA; TBE, Tris-borate-EDTA; TREX1, three prime repair exonuclease 1. (TIF) [file pbio.2005653.s004.tif]

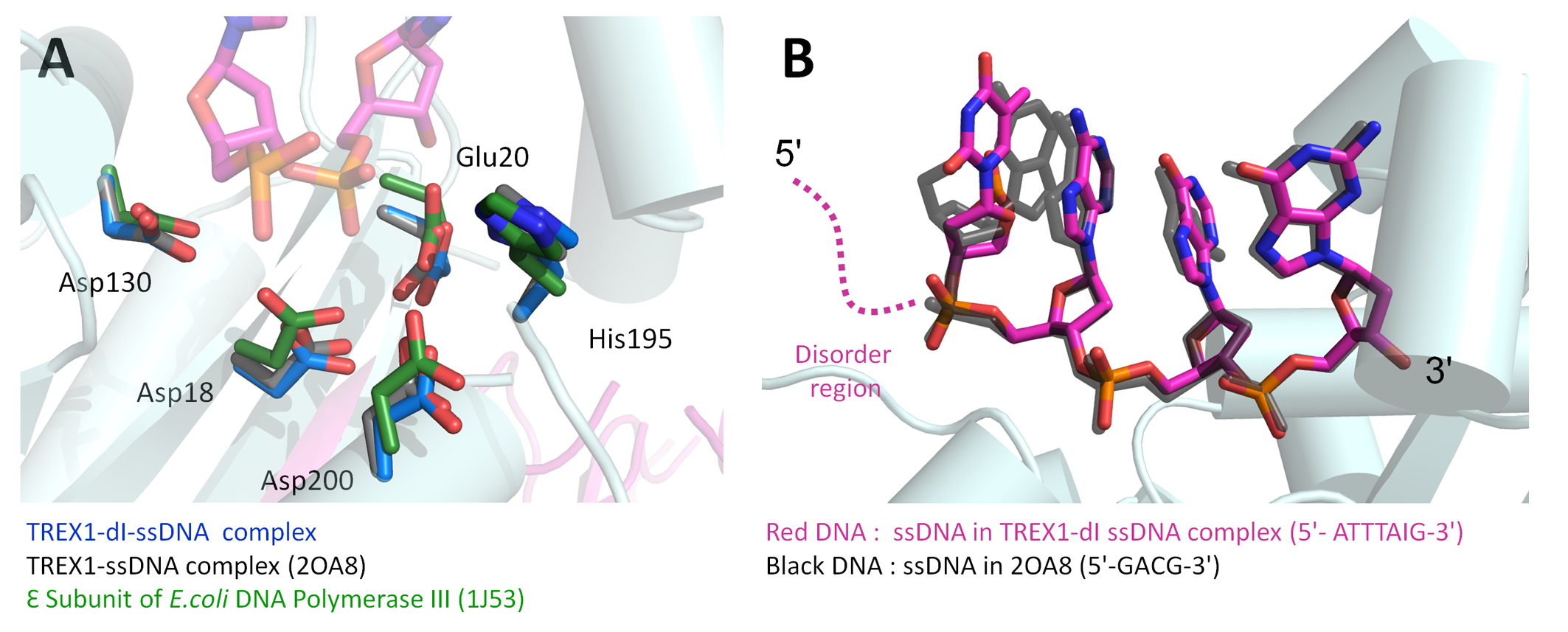

Supplement: S3 Fig — (A) Structural comparison of the active sites in TREX1-dI-ssDNA complex, TREX1-ssDNA complex (PDB Accession Code: 2OA8), and ε subunit of the DNA polymerase (PDB Accession Code: 1J53). (B) Structural comparison of the ssDNA in TREX1-dI-ssDNA and TREX1-ssDNA complex (PDB Accession Code: 2OA8). dI, dioxyinosine; dI-ssDNA, ssDNA containing a dI; PDB, Protein Data Bank; single-stranded DNA; TREX1, three prime repair exonuclease 1. (TIF) [file pbio.2005653.s005.tif]

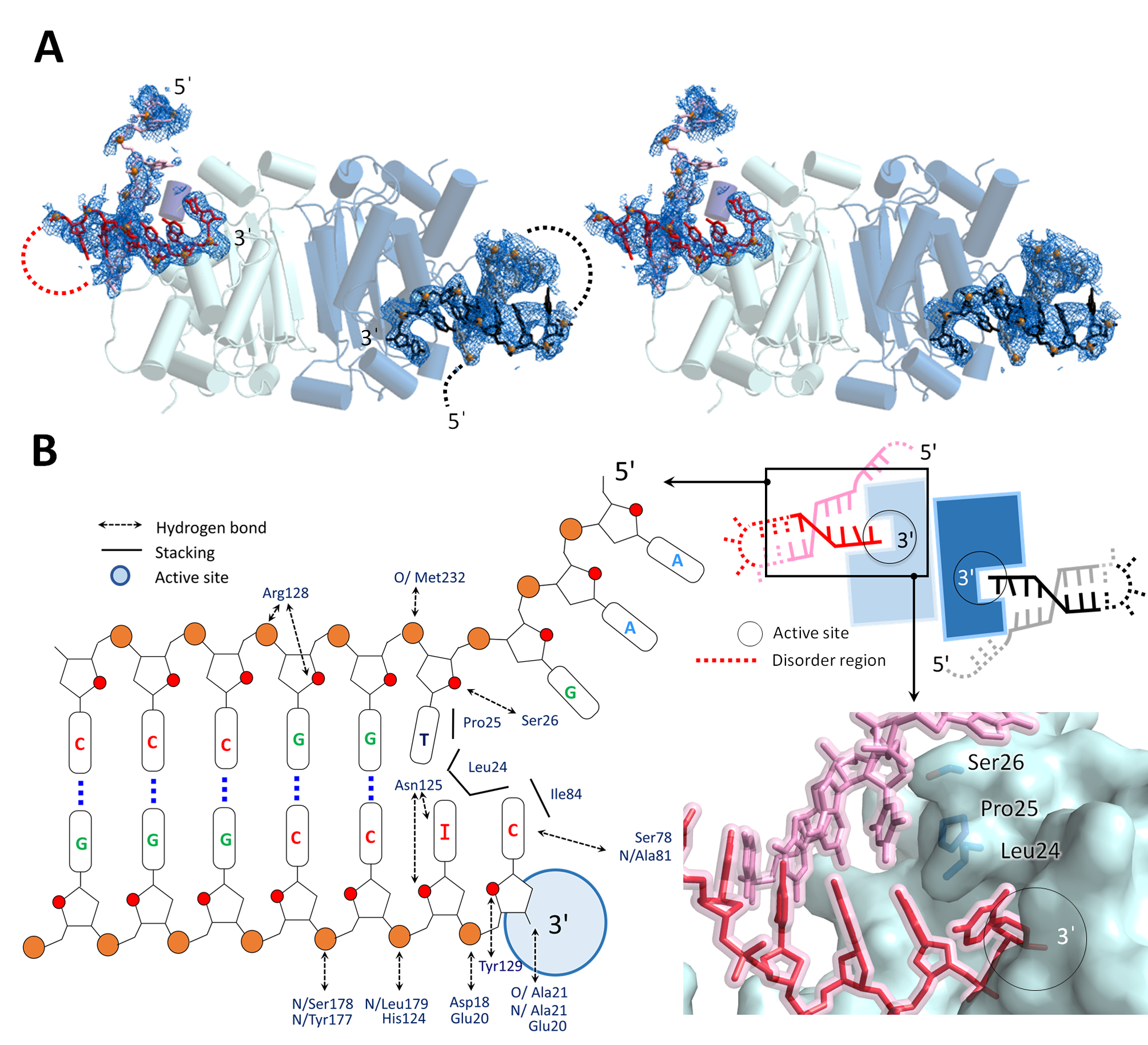

Supplement: S4 Fig — (A) The omitted electron density map (Fo − Fc, 1.0 σ) of 2 dI-dsDNAs in the TREX1-dI-T-dsDNA complex. The DNA with dotted lines indicates the disordered DNA regions in the crystal structure. (B) The upper right panel shows the schematic and overall structure of the TREX1-dI-T-dsDNA complex. The right bottom panel shows the surface of the Leu24-Pro25-Ser26 cluster. The left panel displays the schematic of the interactions between TREX1 and dI-containing dsDNA. dI, dioxyinosine; dI-T-dsDNA, dsDNA with a scissile strand containing a dI; dsDNA, double-stranded DNA; TREX1, three prime repair exonuclease 1. (TIF) [file pbio.2005653.s006.tif]

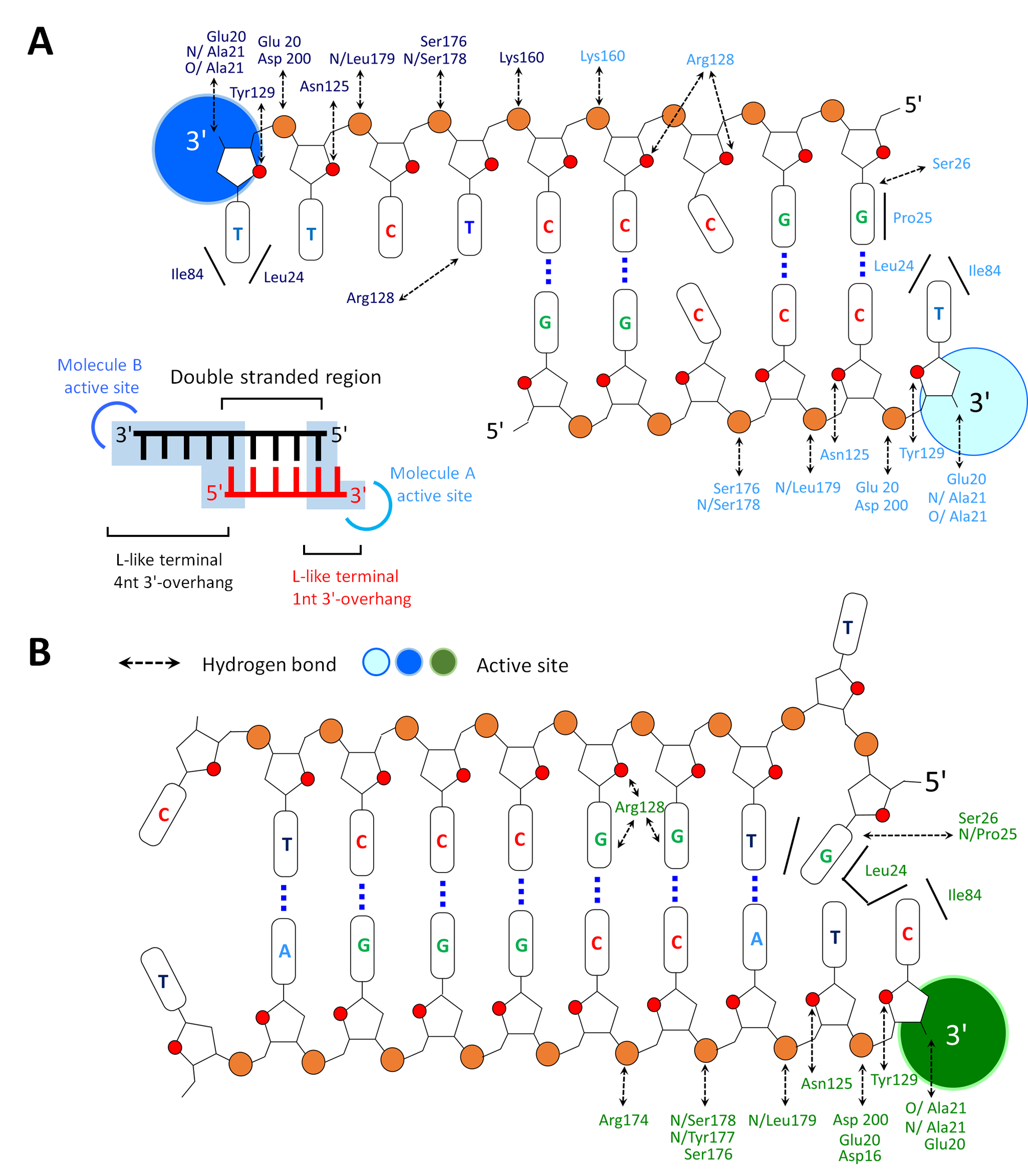

Supplement: S5 Fig — (A) For the structure of the TREX1-L-structural dsDNA complex. (B) For the structure of the TREX1-Y-structural dsDNA complex. dsDNA, double-stranded DNA; TREX1, three prime repair exonuclease 1. (TIF) [file pbio.2005653.s007.tif]

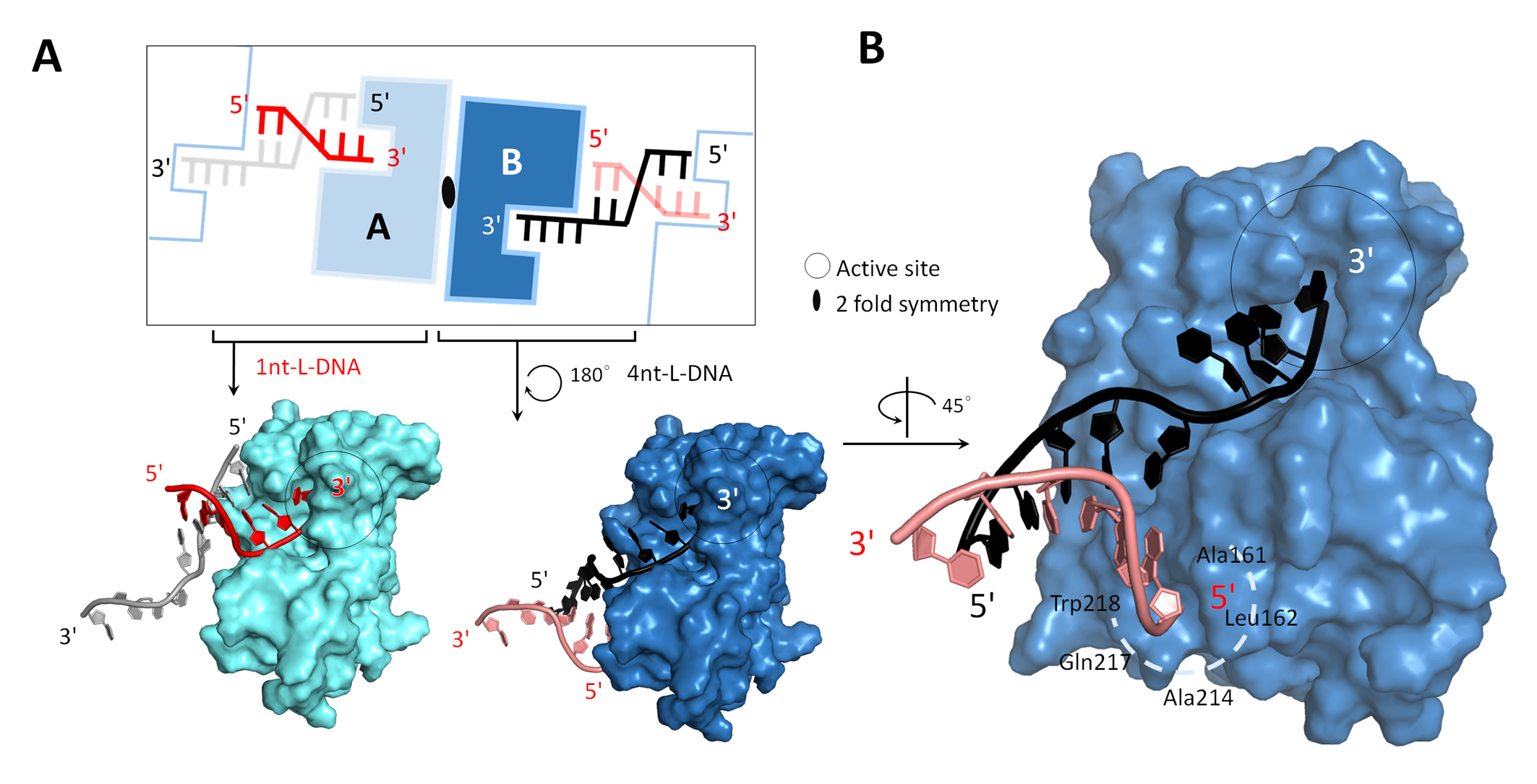

Supplement: S6 Fig — (A) Schematic of the two binding modes for TREX1 with 1-nt-L-DNA and 4-nt-L-DNA. Two 5′-ends of duplex DNA are blocked by different residues of 2 TREX1 protomers. Left and right panels show the blocking regions of TREX1 on 1 and 4 nt 3′-overhang, respectively. (B) Ala161, Leu162, Ala214, Gln217, and Trp218 in molecule B are in contact with the 5′-end of 4 nt L-DNA. However, no hydrogen bonds are formed in this region. TREX1, three prime repair exonuclease 1. (TIF) [file pbio.2005653.s008.tif]

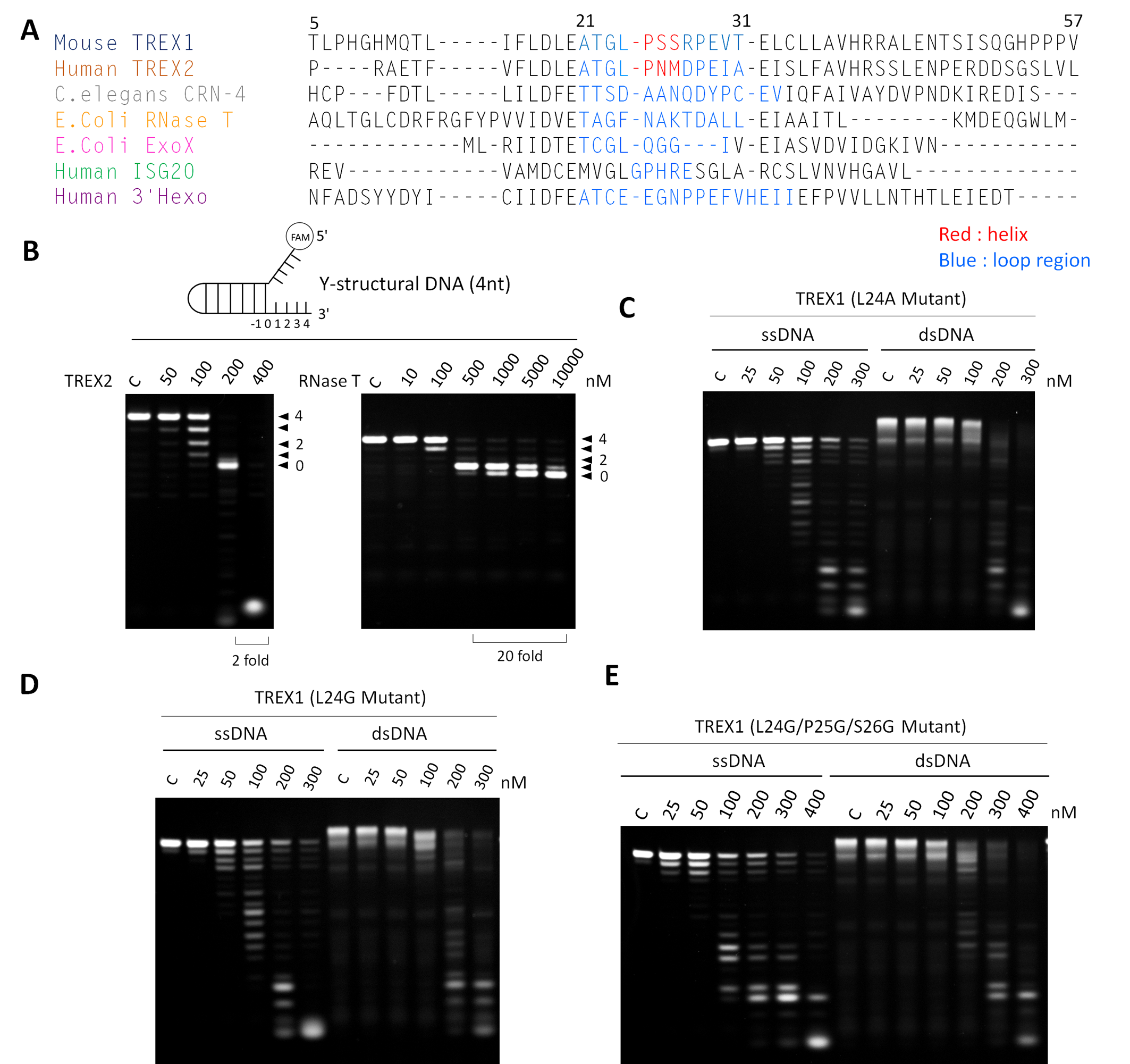

Supplement: S7 Fig — (A) Sequence alignment of TREX1 and other DEDDh exonucleases. The loop and helix regions between β-strand 1 (β1, 12–22 a.a.) and β-strand 2 (β2, 31–40 a.a.) are highlighted in blue and red, respectively. (B) Nuclease activities of TREX2 and RNase T on digesting Y-structural DNA. The structural restriction from a double-stranded structure at RNase T is stronger than that at TREX1 and TREX2. (C) (D) (E) Nuclease activities of 3 TREX1 mutants (L24A, L24G, and L24G/P25G/S26G) on digesting ssDNA and dsDNA. dsDNA, double-stranded DNA; ssDNA, single-stranded DNA; TREX1, three prime repair exonuclease 1; TREX2, three prime repair exonuclease 2. (TIF) [file pbio.2005653.s009.tif]

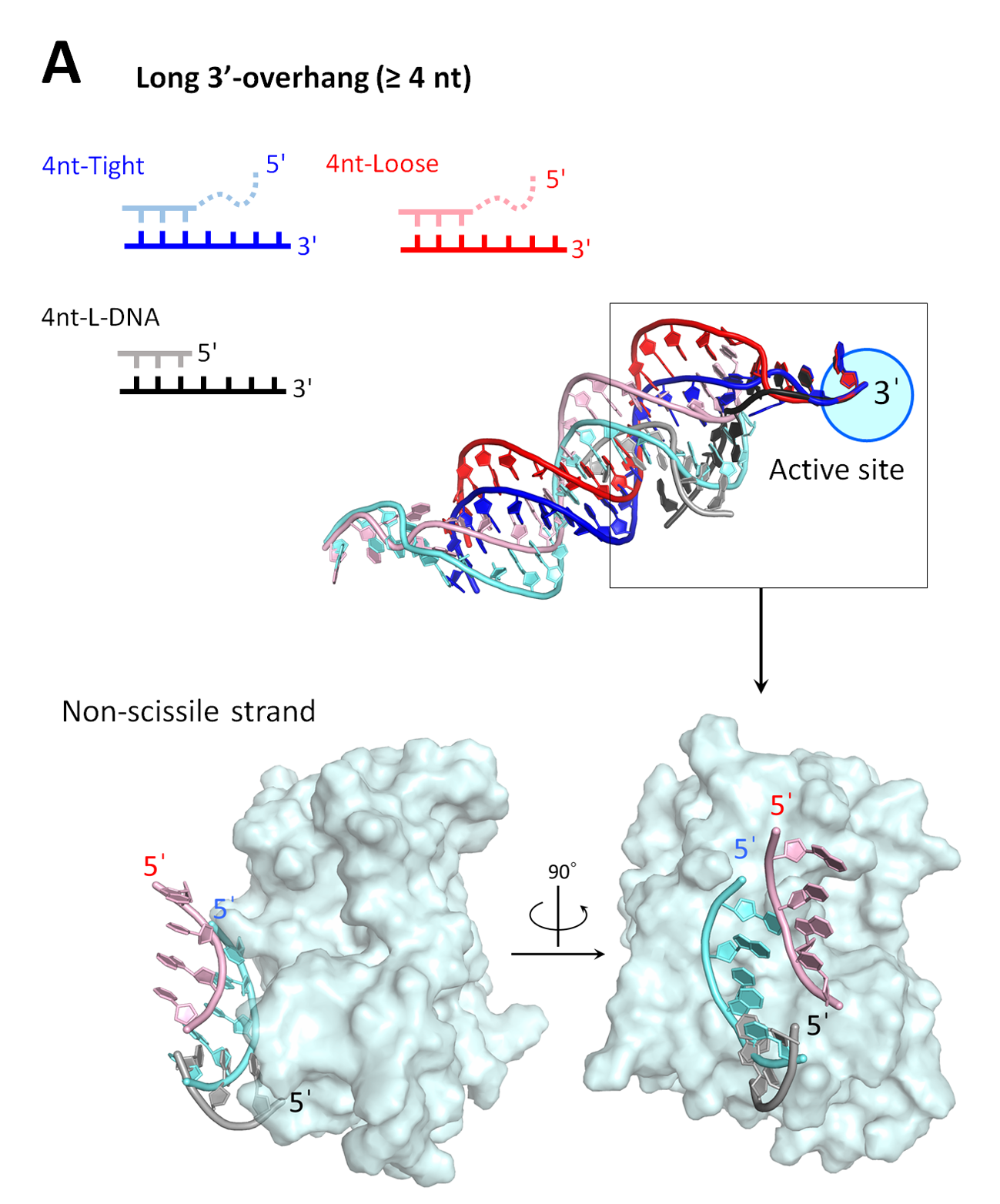

Supplement: S8 Fig — (A) Superposition of the three structures of TREX1 in complex with duplex DNA with long 3′-overhang (≥4-nt), including 4-nt-long 3′-overhang (4 nt-L-DNA) in the TREX1-L-structural dsDNA complex and tight and loose conformations in the previous TREX1-dsDNA complex (PDB accession code: 4YNQ). The colors and schematic diagrams of 3 duplex DNAs are displayed in the top panel. The nonscissile strands of 3 duplex DNAs are in contact with TREX1 in different loci. For clarity, only partial nucleotides at the 5′-end of the nonscissile strands are shown. dsDNA, double-stranded DNA; PDB, Protein Data Bank; TREX1, three prime repair exonuclease 1. (TIF) [file pbio.2005653.s010.tif]

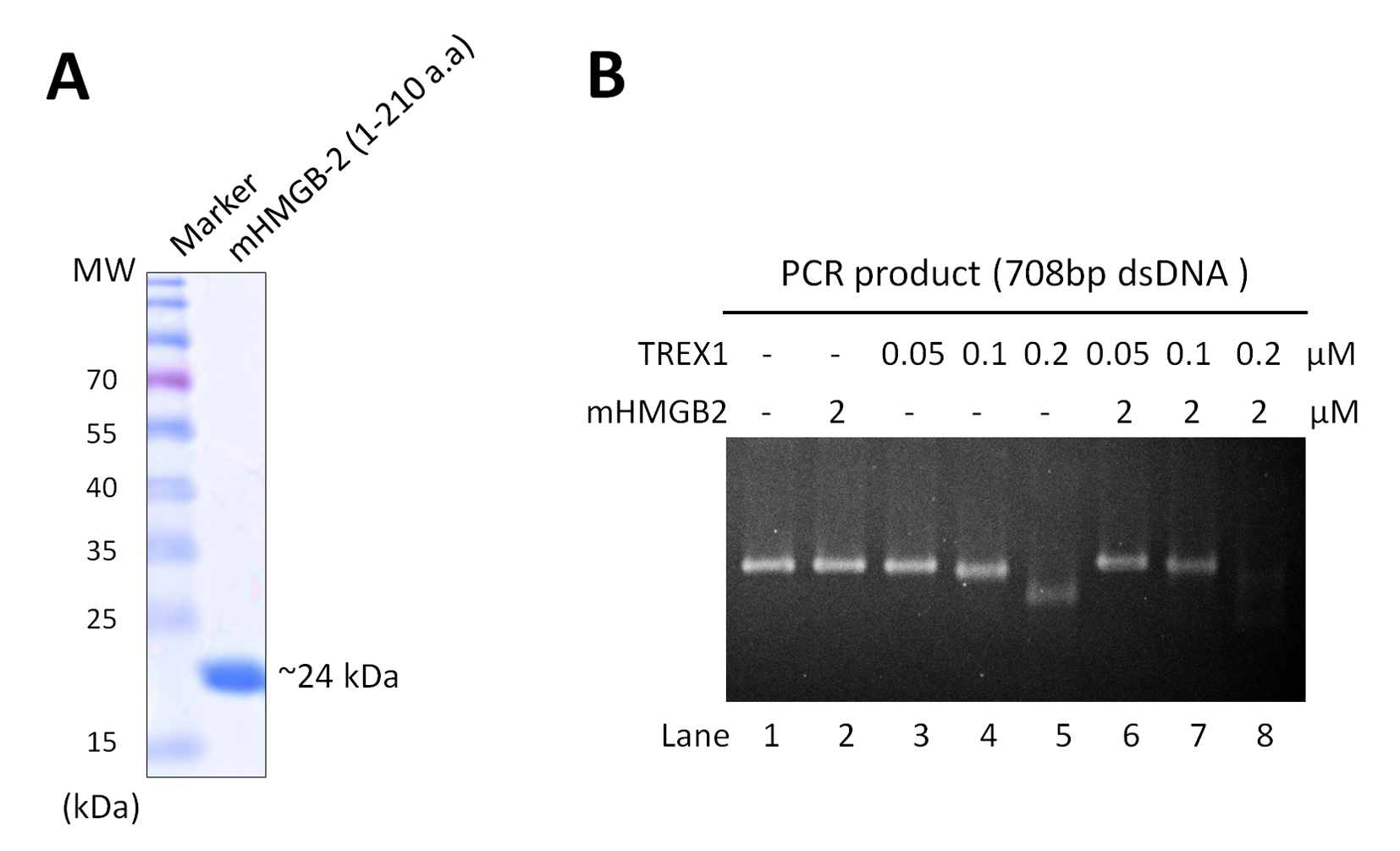

Supplement: S9 Fig — (A) SDS-PAGE analysis of purified mHMGB-2 (1–210 a.a.). (B) Assays of TREX1 DNase activity in the presence or absence of mHMGB-2. A linear 708-bp dsDNA (300 ng) was incubated with mTREX1 and mHMGB-2, and the DNA digests were analyzed by gel electrophoresis. TREX1 cleaved linear dsDNA more efficiently in the presence of mHMGB-2 (by comparing with lane 5 and 8). dsDNA, double-stranded DNA; mHMGB-2, mouse HMGB-2; TREX1, three prime repair exonuclease 1. (TIF) [file pbio.2005653.s011.tif]
